# Local insulin-like growth factor-1 and Akt/mTOR signaling in skeletal muscle of lubabegron-supplemented finishing steers

**DOI:** 10.1093/jas/skag281

**Published:** 2026-09-02

**Authors:** Donghun Kang, Byungkyoo Park, Erika P Eckhardt, Anna M Munger, Andrea M Luttman, Andrea Garmyn, Nathan Pyatt, Jongkyoo Kim

**Affiliations:** Department of Animal Science, Michigan State University, East Lansing, MI 48823, United States; Department of Animal Science, Michigan State University, East Lansing, MI 48823, United States; Department of Animal Science, Michigan State University, East Lansing, MI 48823, United States; Department of Animal Science, Michigan State University, East Lansing, MI 48823, United States; Department of Animal Science, Michigan State University, East Lansing, MI 48823, United States; Department of Animal Science, Michigan State University, East Lansing, MI 48823, United States; Department of Food Science and Human Nutrition, Michigan State University, East Lansing, MI 48823, United States; Elanco Animal Health, Indianapolis, IN 46221, United States; Department of Animal Science, Michigan State University, East Lansing, MI 48823, United States; Department of Food Science and Human Nutrition, Michigan State University, East Lansing, MI 48823, United States

**Keywords:** lubabegron, β-adrenergic ligands, beef steers, skeletal muscle, intramuscular fat

## Abstract

Lubabegron (LUB) is a β-adrenergic agonist/antagonist approved for reduction of ammonia gas emissions in feedlot cattle; improvements in growth and carcass yield have also been reported, although the underlying mechanism remains unresolved. Thirty-two Angus-influenced steers (524 ± 5 kg) were assigned to control (CON) or LUB diets (3.5 mg/kg dry matter [DM]; targeted 36 mg/steer/d for 63 d). All steers had received a trenbolone acetate–estradiol implant (TE-200) at arrival, 58 d before treatment initiation, administered identically across groups. Growth performance, carcass traits, longissimus muscle (LM) composition, transcriptome, signaling protein abundance, and insulin-like growth factor-1 (IGF-1) were measured. LUB increased final body weight (637 vs. 612 kg), average daily gain, dry matter intake, and gain-to-feed ratio relative to CON (*P* < 0.01). Hot carcass weight was 18.5 kg greater in LUB (*P* < 0.01), whereas dressing percentage, REA, 12th-rib fat, and marbling score were unaffected (*P* ≥ 0.25). LM crude protein was higher (23.6% vs. 22.5%; *P* < 0.01) and crude fat tended lower (5.0% vs. 6.4%; *P* = 0.051) in LUB steaks. Warner–Bratzler shear force was greater in LUB at 2 d (4.36 vs. 3.67 kg; *P* = 0.03) and 14 days (3.74 vs. 2.76 kg; *P* < 0.01) postmortem. Serum creatinine, total protein, bilirubin, and cholesterol were elevated in LUB steers (*P* ≤ 0.02). Transcriptomic profiling at d 56 identified 643 differentially expressed genes, with upregulation of myogenic regulators, glycolytic enzymes, and fast-twitch fiber genes and downregulation of adipogenic networks. Phospho-mTOR abundance (*P* = 0.03) and the p-mTOR/mTOR ratio (*P* = 0.05) were greater in LUB. Although LUB is an established β_3_-AR agonist, receptor binding and protein abundance were not assessed; β_3_-AR transcript in LM was minimal and undetectable in 40.6% of samples, and neither p-CREB nor the phosphorylated-cAMP response element-binding protein CREB (p-CREB)/CREB ratio differed between treatments (*P* ≥ 0.32), arguing against p-CREB-dependent β_3_-AR signaling as the primary driver. IGF-1 protein in LM was greater in LUB-supplemented steers at d 56 (*P* = 0.01), whereas circulating IGF-1 did not differ overall (*P* = 0.66), although a treatment × time interaction was detected (*P* < 0.05). The dissociation between elevated local muscle IGF-1 and the absence of a sustained systemic increase supports an autocrine and paracrine IGF-1 mechanism with downstream Akt/mTOR activation as a coherent framework for the muscle response observed with LUB supplementation.

## Teaser

Lubabegron supplementation elevated local insulin-like growth factor-1 (IGF-1) protein within bovine skeletal muscle independently of systemic IGF-1, with activation of the Akt/mTOR axis and concurrent transcriptional remodeling consistent with a shift toward lean tissue and away from lipid deposition.

## Introduction

Skeletal muscle accretion is a principal determinant of productivity and carcass value in finishing beef cattle, governed by coordinated interactions among hormonal signals, nutrient-sensing pathways, and transcriptional programs that regulate satellite cell activity, myofiber hypertrophy, and metabolic adaptation (Wang et al. 2014; Thornton 2019; Du 2023; Gunderson and Anas 2025). β-adrenergic receptor agonists (β-AA) such as ractopamine and zilpaterol have been used to enhance lean tissue accretion during the finishing period (Scramlin et al. 2010). Their mechanism of action is well characterized: agonist binding to β_1_- and β_2_-adrenergic receptors (β_1_/β_2_-AR) activates adenylyl cyclase, elevates intracellular cyclic adenosine monophosphate (cAMP), and stimulates protein kinase A (PKA). Downstream PKA signaling drives cAMP response element-binding protein (CREB)-mediated transcriptional activation and Akt/mTOR-dependent protein synthesis while attenuating protein degradation, producing a net anabolic response (Anderson et al. 2005; Johnson et al. 2014).

Lubabegron (LUB; Experior®, Elanco Animal Health) is a β-adrenergic modulator with a pharmacological profile distinct from conventional β-AA. It was approved by the U.S. Food and Drug Administration (FDA) in 2018 (NADA 141-508) for the reduction of ammonia gas emissions in beef steers and heifers fed in confinement for slaughter during the last 14–91 d on feed (FDA 2018). This emission-reduction effect is linked to altered nitrogen retention and nutrient partitioning in finishing cattle, and the skeletal muscle consequences of that partitioning are the basis for the present mechanistic investigation. Whereas ractopamine and zilpaterol function as β_1_/β_2_-AR agonists, LUB acts as a selective β_3_-AR agonist with concurrent antagonist activity at β_1_- and β_2_-AR subtypes (Dilger et al. 2021; Hwang et al. 2021). Despite this divergent pharmacology, large-scale feedlot trials have consistently reported that LUB supplementation improves average daily gain (ADG), gain-to-feed ratio, and hot carcass weight (HCW), with concurrent reductions in ammonia emissions and, in several studies, marbling score (Kube et al. 2021; Teeter et al. 2021; Edmonds et al. 2024; Kayser et al. 2024). The reproducibility of this response across production environments raises a fundamental mechanistic question: through what molecular pathway is skeletal muscle growth associated with LUB supplementation if not through direct β_1_/β_2_-AR activation?

One proposed explanation invokes β_3_-AR signaling within skeletal muscle. Compared with β_1_- and β_2_-AR subtypes, β_3_-AR is relatively resistant to agonist-induced desensitization, because it lacks the carboxyl-terminal serine and threonine residues that mediate G protein-coupled receptor kinase and β-arrestin-dependent internalization of β₁- and β₂-AR, which could sustain cAMP-PKA signaling under chronic stimulation and at low receptor densities (Nantel et al. 1993; Evans et al. 1996). On this basis, Dilger et al. (2021) suggested that β_3_-AR activation may contribute to skeletal muscle hypertrophy. However, β_3_-AR expression in bovine longissimus muscle has not been systematically characterized, and a direct mechanistic link between β_3_-AR signaling and mTOR-dependent protein accretion in this tissue has not been experimentally established. Furthermore, simultaneous antagonism of β_1_- and β_2_-AR, the predominant β-AR subtypes in skeletal muscle, would be expected to suppress rather than stimulate adrenergic signaling under classical assumptions, rendering direct adrenergic engagement insufficient to account for the magnitude of the muscle response consistently observed in LUB-supplemented cattle. The molecular basis of the muscle accretion associated with LUB supplementation therefore remains undefined.

We hypothesized that the skeletal muscle response to LUB occurs through a mechanism independent of classical β_1_/β_2_-AR signaling, and we sought to identify the upstream signals coupling LUB supplementation to PI3K/Akt/mTOR activation. The specific objectives of this study were to (1) characterize the temporal transcriptional and signaling responses of bovine longissimus muscle to LUB supplementation during the finishing period; (2) evaluate the functional status of the Akt/mTOR axis and upstream adrenergic signaling markers in skeletal muscle.

## Materials and methods

The Michigan State University (MSU) Institutional Animal Care and Use Committee (IACUC; protocol number 202400056) approved all animal procedures and sampling protocols for this study.

### Study animals and pretrial management

#### Animal sourcing and screening

Thirty-two Angus-influenced crossbred steers (524.1 ± 4.9 kg) were used in a 63-d finishing study conducted at the MSU Beef Cattle Teaching and Research Center (BCTRC) in Lansing, MI, United States. Steers were sourced from a commercial backgrounding operation located in Columbia, TN, United States.

To allow for pre-study screening of health status and performance, a total of 63 steers were initially procured. Upon arrival at BCTRC, all steers were processed according to standard feedlot protocols, which included individual identification, body weight measurement, vaccination against respiratory pathogens, antiparasitic treatment, and administration of a trenbolone acetate–estradiol implant (TE-200; Elanco Animal Health, Indianapolis, IN, United States). All steers received a single trenbolone acetate–estradiol implant (TE-200; Elanco Animal Health, Indianapolis, IN, United States) at arrival processing on October 20, 2024, before selection into the trial and before dietary treatment allocation. Implant administration was therefore uniform across all animals and independent of dietary treatment. Dietary treatment began on December 16, 2024, at which point steers were 58 days post-implant. Steers were 85 and 113 days post-implant at day 28 and day 56 biopsies, respectively, and 120 days post-implant at harvest.

Steers were then housed in slatted-floor confinement pens (3.05 × 4.88 m), with four steers per pen.

A finishing diet (Table 1) was introduced using two step-up diets to facilitate dietary adaptation. Once steers reached the target body weight (BW) for the finishing phase, 32 animals with uniform BW and no health abnormalities were selected and enrolled in the study.

**Table 1:** Ingredient composition and nutrient analysis of the finishing diet (dry matter basis).

Items	CON	LUB
**Ingredient, % DM**		
** High moisture corn**	54.24	54.24
** DDGS**	24.00	24.00
** Corn silage**	15.41	15.41
** Supplement 500^1^**	6.35	–
** Supplement 500 + LUB premix^2^**	–	6.35
**Analyzed composition**		
** DM, %**	58.3	58.3
** Crude protein, %**	13.8	13.8
** Crude fat, %**	4.7	4.7
** Ash, %**	5.4	5.4
** NDF, %**	18.1	18.1
** ADF, %**	10.7	10.7
** TDN, %**	80.0	80.0
** NEm, Mcal/kg**	1.97	1.97
** NEg, Mcal/kg**	1.33	1.33

DDGS = distillers dried grains with solubles; NDF = neutral detergent fiber; ADF = acid detergent fiber; TDN = total digestible nutrients; NEm = net energy for maintenance; NEg = net energy for gain; Mcal = megacalorie.

1 CON supplement: vitamin-mineral premix containing monensin at 500 mg/kg DM; manufactured by Kent Nutrition Group (Mason, MI, United States).

2 LUB supplement: same vitamin-mineral premix base as CON, with lubabegron (Experior® Type A medicated article; Elanco Animal Health, Indianapolis, IN, United States) incorporated to produce a Type B medicated article at a final dietary concentration of 3.5 mg/kg DM, delivering a target intake of 36 mg lubabegron per steer per day.

#### Treatment assignment

Approximately 1 week before treatment initiation, BW was recorded on two consecutive mornings before feeding. These pretrial BW measurements were used to allocate the 32 selected steers to eight pens, with four steers per pen, so that BW distributions were comparable among pens. The eight pens were then randomly assigned to one of two dietary treatments, with four pens per treatment, and steers were allowed a 7-d period of pen adaptation before treatment initiation. Dietary treatment was applied at the pen level, and no blocking factor was used:

1. CON (Control [non-treated group]): basal diet without LUB supplementation
2. LUB: basal diet supplemented with LUB at 3.5 mg/kg

Body weight was measured again on two consecutive days immediately before d 0 to establish initial BW and confirm that the treatment groups were similar in initial BW (*P* > 0.10). Interim BW was recorded on d 28 and 56, and final BW was recorded on 2 consecutive days at the conclusion of the 63-day feeding period before transport to the abattoir. Because the steers were maintained under routine feeding management before treatment allocation, no additional fasting period was imposed for BW measurement. To maintain a consistent measurement protocol throughout the study, all BW measurements were collected in the morning before feeding and reported on an unshrunk basis. The same calibrated platform scale (Tru-Test HD5T load bars and ID5000 scale indicator; DATAMARS Livestock, Temple, TX, United States) was used for all measurements. Initial and final BW were calculated as the mean of the 2 consecutive-day measurements collected at the beginning and end of the treatment period, respectively. ADG was calculated as (final BW − initial BW)/63 days, and gain-to-feed ratio (G:F) was calculated on a pen basis as total pen BW gain divided by total pen dry matter intake (DMI).

#### Diet formulation and supplementation

Steers were offered an ad libitum total mixed ration (TMR) formulated on a dry matter basis, consisting of high-moisture corn (54.2%), dried distillers’ grains with solubles (dried distillers’ grains with solubles [DDGS]; 24.0%), corn silage (15.4%), and a vitamin-mineral supplement containing monensin (6.4%) (Table 1). Both treatment supplements were manufactured by Kent Nutrition Group (Mason, MI, United States) as a vitamin-mineral premix base containing monensin at 500 mg/kg. The CON supplement contained no additional active ingredient, whereas the LUB supplement incorporated LUB (Experior®; Elanco Animal Health, Indianapolis, IN, United States) at a final dietary concentration of 3.5 mg/kg DM to deliver a target intake of 36 mg per steer per day.

The resulting premixes were delivered to the MSU feedlot and mixed into the TMR prior to daily feeding. The LUB inclusion rate was designed to deliver a target intake of 36 mg LUB per steer per day.

#### Feed management

A clean bunk management system was implemented, wherein cattle were fed once daily at 0800 h, and bunks were managed to minimize orts. Orts were subsequently collected and weighed on a pen basis each day to estimate feed consumption. To determine dry matter content, weekly samples of the TMR were dried at 105 °C for 24 h in a forced-air oven (DNF600; Yamato Scientific America Inc., Santa Clara, CA, United States). Subsamples were stored at −20°C until submission for proximate analysis via wet chemistry (Dairy One Forage Laboratory, Ithaca, NY, United States). To verify LUB concentration, samples of the premix and TMR were collected on d 1, 28, and 56 and submitted to Eurofins Animal Health Testing (Indianapolis, IN, United States) for analysis. Analyzed LUB concentrations in the premix were 46.0, 56.4, and 51.1 g/ton on d 1, 28, and 56, corresponding to 79%, 97%, and 88% of the formulated concentration, and the corresponding analyzed TMR concentrations were 1.9, 3.5, and 2.6 mg/kg DM. Using the analyzed TMR concentrations together with concurrent pen DMI, estimated LUB intake averaged 35.3 mg/steer/day at d 28 and 27.8 mg/steer/day at d 56 (mean, 31.5 mg/steer/day), or approximately 88% of the 36 mg/steer/day target. The lower analyzed concentration in the d 1 TMR sample, obtained at the start of the supplementation period, and the variation among sampling points are acknowledged as limitations of dose delivery.

### Biological sample collection and processing

#### Blood collection

Blood samples were collected via jugular venipuncture on d 0, 28, and 56 of the trial, before the morning feeding. Sampling was performed using an 18-gauge × 1.5-inch needle (Greiner Bio-One North America Inc., Monroe, NC, United States), and blood was drawn into evacuated serum tubes (Dickinson and Company, Franklin Lakes, NJ, United States). After collection, samples were allowed to clot at room temperature for 30 min, followed by centrifugation at 3,500 × *g* for 15 min to separate the serum. Serum aliquots were then transferred into sterile 1.5 mL microcentrifuge tubes and stored at −20°C until further analysis.

Serum samples (*n* = 32 per time point) were submitted to the MSU Veterinary Diagnostic Laboratory (MSU VDL), Clinical Pathology Section, for a comprehensive bovine serum chemistry panel. Analytes included urea nitrogen, creatinine, sodium, potassium, chloride, total CO₂ (TCO₂), sodium-to-potassium ratio (Na:K), anion gap, calculated osmolarity, calcium, phosphorus, magnesium, total protein, albumin, calculated globulin, glucose, total bilirubin, indirect bilirubin, alkaline phosphatase (ALP), gamma-glutamyl transferase (GGT), aspartate aminotransferase (AST), creatine kinase (CK), and cholesterol.

A subset of serum samples was used to quantify circulating LUB concentrations using ultra-performance liquid chromatography–tandem mass spectrometry (UPLC–MS/MS). Approximately 50 µL of serum was mixed with 150 µL of methanol containing 10 nM telmisartan as an internal standard. The mixture was vortexed and centrifuged at 10,000 × *g* for 5 min to precipitate proteins. A standard calibration curve for LUB was prepared over a concentration range of 0.24 nM to 1 µM, using 10 nM telmisartan as the internal standard. Quantitative analysis was performed on a Waters Xevo TQ-S micro UPLC/MS/MS system (Waters Corporation, Milford, MA, United States). A 2 µL aliquot of the supernatant was injected and separated using an 8-min linear gradient on a Supelco Ascentis Express C18 column (2.1 × 50 mm, 2.7 µm particle size). The mobile phases consisted of solvent A (0.1% formic acid in water) and solvent B (methanol: acetonitrile, 1:1 vol/vol, with 0.1% formic acid). The gradient program was as follows: 10% B from 0.00 to 1.00 min; a linear increase to 99% B from 1.00 to 5.00 min; hold at 99% B from 5.00 to 6.00 min; and return to 10% B from 6.01 to 8.00 min. The flow rate was maintained at 0.3 mL/min, and the column temperature was set at 40 °C. The mass spectrometer operated in positive electrospray ionization mode, and data were acquired using multiple reaction monitoring. Source parameters included a capillary voltage of 1.0 kV, source temperature of 150 °C, desolvation temperature of 350 °C, desolvation gas flow of 800 L/h, and cone gas flow of 40 L/h.MS/MS parameters for LUB and Telmisartan are listed in Table S1.

#### Longissimus muscle biopsies and analysis

Skeletal muscle biopsy samples were collected from the longissimus muscle (LM) between the 12th and 13th ribs on d 0, 28, and 56 of the treatment periods. Biopsies were performed using a custom-made 6 mm Bergström needle (Samyoung, South Korea), following modified procedures described by Smith et al. (2019) and Eckhardt et al. (2024).

Steers were restrained in a squeeze chute during the procedure to ensure animal safety and minimize stress. The biopsy site was prepared by removing hair, followed by disinfection using povidone-iodine and 70% ethanol. Local anesthesia was administered via subcutaneous injection of 15 mL of 2% lidocaine (Covetrus, Portland, ME, United States). A small incision (approximately 1 cm) was made with a sterile scalpel, and >0.5 g of muscle tissue was collected using the biopsy needle. To minimize blood contamination, efforts were made to obtain a complete sample on the first attempt. If necessary, a second pass was made, and the collected tissue was gently blotted to remove any residual blood. On d 0 and 56, biopsies were taken from the left side of each steer, avoiding previous biopsy sites, while samples on d 28 were obtained from the right side. Following the biopsy, the site was treated with a topical antiseptic and monitored to ensure proper healing. Samples were immediately snap-frozen in liquid nitrogen, transported on dry ice to the MSU Muscle Biology Laboratory, and stored at −80°C until further analysis.

#### Carcass data collection

Final BW was recorded at BCTRC on the morning of transport prior to loading; no shrink BW was collected at the abattoir. Steers were transported approximately 213 km to a federally inspected commercial abattoir in Falmouth, MI, United States, held overnight without feed, and harvested the following morning under United States Department of Agriculture (USDA) supervision. Hot carcass weight was recorded immediately after evisceration.

Following a 48-h chilling period, trained personnel from MSU collected carcass measurements. For each of the 32 carcasses, data collected included HCW, REA, 12th-rib fat thickness, percentage of kidney, pelvic, and heart fat (KPH%), marbling score, and USDA quality grade. The USDA yield grade was calculated based on the official equation (Morgan 1997). Personnel conducting carcass grading, proximate composition, Warner–Bratzler Shear Force (WBSF), and molecular analyses were blinded to treatment assignment.


USDA YG=2.5+(2.5×adjusted fat thickness, inches)+(0.2×KPH)+(0.0038×HCW, pounds)−(0.32×REA, sq. in.)

Instrumental color measurements were collected from three distinct regions of the exposed left LM surface after a minimum bloom time of 30 min and averaged. Color was evaluated in the CIE Lab* color space, including lightness (L*), redness (a*), and yellowness (b*), using a colorimeter (Nix Pro 2 Color Sensor, Nix Sensor Ltd., Hamilton, Ontario, Canada). CIELAB reference values were obtained using D65 illumination and a 2° observer angle. Ultimate pH was measured at the LM surface 48 h postmortem using a portable meat pH meter (Hanna Instruments, Smithfield, RI, United States).

#### Postmortem evaluation of muscle characteristics

Following carcass data collection, an 8.89 cm section from the anterior end of the left LM (LM refers specifically to the longissimus lumborum in postmortem analyses) was excised and transported to the MSU Meat Laboratory. The loin section was then fabricated into two steaks, each 2.54-cm thick, which were randomly assigned to postmortem aging periods of 2 or 14 d. An additional portion of the left LM was collected for compositional analysis, ensuring a minimum of 200 g of muscle tissue per carcass.

All steaks and compositional samples were vacuum packaged. Steaks designated for 2-d aging and all compositional analysis samples were stored at −20°C. Steaks assigned to the 14-d aging period were stored at 2–4°C, then transferred to −20°C until further analysis.

#### Chemical composition analysis

Muscle samples (200 g) designated for compositional analysis from each carcass were cut into small cubes and homogenized. Proximate composition, including percentages of fat, moisture, and protein, was determined using a near-infrared spectrophotometer (FoodScan; FOSS NIRSystems, Inc., Laurel, MD, United States) following AOAC Official Methods *2007.04* (Anderson et al. 2007).

#### Cooking loss and WBSF analysis

Following completion of their assigned postmortem aging periods (2 or 14 d), steaks were cooked to evaluate cooking loss and WBSF. Prior to cooking, each steak was weighed to determine raw weight. Steaks were then cooked on a press grill (Model GR-150P1; Conair, East Windsor, NJ, United States) until reaching an internal temperature of 70 °C, monitored using a thermocouple thermometer (Model OM-HL-EH-TC; Omega Engineering). Upon reaching the target temperature, steaks were removed from the grill, rested for 2 min at room temperature, and reweighed to calculate cooking loss.

Cooked steaks were placed on individual polystyrene foam trays, overwrapped with polyvinyl chloride film, and stored at 3 °C for 24 h. After chilling, six cores (1.3 cm diameter) were extracted from each steak parallel to the muscle fiber orientation using a drill-mounted coring device. Cores were then sheared perpendicular to the muscle fibers using a texture analyzer (TA.XT Texture Analyzer Stable Micro Systems Ltd., Godalming, UK) fitted with a V-shaped Warner–Bratzler blade at a crosshead speed of 200 mm/min.

#### Total RNA extraction and analysis

Total RNA was isolated from approximately 0.1 g tissue samples using acid guanidinium thiocyanate–phenol–chloroform extraction reagent (TRIzol™ reagent; ThermoFisher Scientific) and two sterilized 5-mm stainless steel beads. Homogenization was performed using a bead mill homogenizer (TissueLyser System II; QIAGEN, Hilden, Germany) at 180 Hz for 4 min. RNA extraction followed the protocol described by Eckhardt et al. (2024), with modifications including chloroform-based phase separation, isopropanol precipitation, and ethanol washing. RNA pellets were resuspended in diethylpyrocarbonate-treated water and dissolved at 60 °C. RNA quality and concentration were assessed using a UV-Vis spectrophotometer (NanoDrop One/OneC, ThermoFisher Scientific).

Genomic DNA was removed, and cDNA was synthesized using the High-Capacity cDNA Reverse Transcription Kit (ThermoFisher Scientific), following the manufacturer’s instructions. The resulting cDNA was diluted to 100 ng/µL and stored at −20°C until use.

Gene expression was quantified by quantitative reverse transcription polymerase chain reaction (RT-qPCR). Quantitative PCR was performed on the resulting cDNA using a real-time PCR system (QuantStudio 6 Pro; ThermoFisher Scientific). To ensure consistent quantification across all samples, gene expression was normalized to two housekeeping genes: ribosomal protein S9 (*RPS9*) and hydroxymethylbilane synthase (*HMBS*). The average cycle threshold (Ct) value of *RPS9* and *HMBS* was used as the endogenous control to calculate relative target gene expression. Genes of interest within this study include myogenic factor 5 (*MYF5*), myogenic differentiation (*MYOD1*), myogenin (*MYOG*), insulin-like growth factor 1 (*IGF1*), myosin heavy chain (*MHC*) types *I, IIA*, and *IIX*, β-adrenergic receptor (*AR*) *beta 1* (*ARB1*)*, AR* beta 2 (*ARB2*)*, AR* beta 3 (*ARB3*), CCAAT/enhancer-binding protein alpha (*C/EBPα*), peroxisome proliferator-activated receptor alpha (*PPARα*), peroxisome proliferator-activated receptor gamma (*PPARγ*), peroxisome proliferator-activated receptor gamma coactivator-1 alpha (*PGC-1α*), cyclic AMP response element-binding protein (*CREB*), fatty acid binding protein 4 (*FABP4*), zinc finger protein 423 (*ZNF423*), TGF-beta activated kinase 1 (*TAB1*), and collagen type I alpha 1 (*COL1A1*). Gene expression was quantified using the Applied Biosystems TaqMan Fast Advanced Master Mix (ThermoFisher Scientific) and TaqMan Gene Expression Assays (ThermoFisher Scientific; Table 2). All assays were conducted in duplicate, and thermal cycling conditions were set according to the manufacturer’s recommendations (45 cycles of 15 s at 95 °C and 1 min at 60 °C). RT-qPCR was performed on LM biopsy samples collected at d 0, 28, and 56 from all 32 steers, and treatment effects were evaluated as the change from the d 0 baseline at d 28 and 56.

**Table 2:** List of TaqMan assay and primer sequences for RT-qPCR assays in skeletal muscle samples.

Gene symbol	TaqMan assay ID	Manufacturer^1^	Target description
***RPS9***	Bt03272016_m1	ThermoFisher	Ribosomal protein^2^
***HMBS***	Bt03234763_m1	ThermoFisher	Hydroxymethylbilane synthase^2^
***MYF5***	Bt03223134_m1	ThermoFisher	Myogenic factor 5
***MYOD1***	Bt03244740_m1	ThermoFisher	Myoblast determination protein
***MYOG***	Bt03258929_m1	ThermoFisher	Myogenin
***IGF1***	Bt03252282_m1	ThermoFisher	Insulin-like growth factor 1
***ADRB1***	Bt03224845_s1	ThermoFisher	Beta-1 adrenergic receptor
***ADRB2***	Bt03291916_s1	ThermoFisher	Beta-2 adrenergic receptor
***ADRB3***	Bt03213472_m1	ThermoFisher	Beta-3 adrenergic receptor
***C/EBPα***	Bt03224529_m1	ThermoFisher	CCAAT/enhancer-binding protein alpha
***PPARα***	Bt03220821_m1	ThermoFisher	Peroxisome proliferator-activated receptor alpha
***PPARγ***	Bt03217547_m1	ThermoFisher	Peroxisome proliferator-activated receptor gamma
***PGC-1α***	Bt01208835_m1	ThermoFisher	Peroxisome proliferator-activated receptor gamma coactivator 1-alpha
***CREB***	Bt03213703_m1	ThermoFisher	Cyclic AMP response element-binding protein
***FABP4***	Bt03213820_m1	ThermoFisher	fatty acid binding protein 4
***ZNF423***	Bt02721988_m1	ThermoFisher	Zinc finger protein 423
***TAB1***	Bt03266423_m1	ThermoFisher	TGF-beta activated kinase 1
***COL1A1***	Bt01463861_g1	ThermoFisher	Collagen type I alpha 1
***MYH7***	Bt03224257_m1	ThermoFisher	Myosin heavy chain I
**Gene**	Primer and probe sequence (5ʹ to 3ʹ)		
***MYH2***		ThermoFisher	Myosin heavy chain IIA
** Forward**	GCAATGTGGAAACGATCTCTAAAGC		
** Reverse**	GCTGCTGCTCCTCCTCCTG		
** Probe**	6FAM-TCTGGAGGACCAAGTGAAC GAGCTGA-TAMRA		
***MYH1***		ThermoFisher	Myosin heavy chain IIX
** Forward**	GGCCCACTTCTCCCTCATTC		
** Reverse**	CCGACCACCGTCTCATTCA		
** Probe**	6FAM-CGGGCACTGTGGACTACA ACATTACT-TAMRA		

1 This table summarizes the TaqMan® assays used for quantifying gene expression. All assays were purchased from ThermoFisher Scientific and designed for use with *Bos taurus* gene targets.

2 Reference genes.

#### RNA sequencing

For RNA sequencing, total RNA was isolated from a silica-membrane-based RNA extraction kit (RNeasy Mini Kit; Qiagen). The integrity of isolated RNA was assessed using a microfluidic electrophoresis system (Agilent 4200 TapeStation), and only samples with an RNA Integrity Number (RIN) ≥ 7.0 were submitted for mRNA sequencing to the MSU Research Technology Support Facility Genomics Core.

mRNA libraries were prepared using a commercial library preparation kit (Watchmaker Genomics) with dual-index primers and sequenced in a 2 × 150 bp paired-end format on the AVITI system (Element Biosciences). Raw reads were demultiplexed, converted to FastQ format, and processed for quality control, adapter removal, and trimming using standard tools (FastQC, Trimmomatic, and TrimGalore). High-quality paired reads were aligned to the *Bos taurus* reference genome (ARS-UCD 1.3) using HISAT2, and gene-level counts were generated with HTSeq.

Differential expression analysis was performed using DESeq2. A linear model was constructed to compare treated vs. control cattle at d 56, corrected for d 0 differences. Genes with a baseMean < 100 were excluded, and differentially expressed genes (DEGs) were defined as those with a false discovery rate (FDR) < 0.1. DEGs were subjected to gene set enrichment analysis (GSEA) using g:Profiler (g:GOst) restricted to annotated *B. taurus* genes. The FDR < 0.1 threshold was selected a priori as an exploratory discovery criterion appropriate for a hypothesis-generating transcriptomic screen with modest biological replication, prioritizing detection of coordinated, pathway-level signals over strict per-gene control of false positives. Candidate genes central to the proposed mechanism were subsequently validated by RT-qPCR.

#### Protein extraction and Western blot analysis

Biopsy samples from the left LM collected at d 0 and 56 and stored at −80°C were used for total protein extraction. Approximately 0.1 g of tissue was homogenized in a bead mill homogenizer using a detergent-based lysis buffer (T-PER; ThermoFisher Scientific) containing protease inhibitors. Lysates were clarified by centrifugation, and protein concentrations were quantified using a Pierce™ BCA Protein Assay Kit (ThermoFisher Scientific, Rockford, IL, United States). Extracted proteins were diluted to 2 µg/µL and stored at −80°C until Western blotting.

For Western blot (WB) analysis, equal amounts of protein were denatured, separated by SDS-PAGE using appropriate low- and high-molecular-weight gradient polyacrylamide gels, and transferred to nitrocellulose membranes using a dry blotting system. Membranes were stained to confirm protein transfer, blocked in either 5% non-fat milk or 5% BSA (for phospho-proteins), and probed with primary antibodies targeting mTOR, phospho-mTOR, Akt, phospho-Akt, p70^s6k^, phospho-p70^s6k^, insulin-like growth factor 1 (IGF-1), PGC-1α, CREB, phospho-CREB, AMP-activated protein kinase (AMPK), and phospho-AMPK (see Table S2 for antibody details). HRP-conjugated secondary antibodies were used, and detection was performed using a chemiluminescent substrate and visualized with a digital imaging system (iBright 1500; ThermoFisher Scientific).

### Mitochondrial DNA (mtDNA)

Total DNA was isolated from LM biopsy samples using a commercial genomic DNA purification kit (Wizard® Genomic DNA Purification Kit; Promega, Madison, WI, United States). mtDNA abundance, expressed as the mtDNA-to-nuclear DNA ratio, was assessed by qPCR using total DNA as template and primers targeting cytochrome c oxidase subunit II (*COX2*) as the mitochondrial marker and β-actin (*ACTB*) as the nuclear reference (Kang et al. 2026). Primer and probe sequences were designed from bovine genomic DNA using Primer Express™ v3.0.1 (Applied Biosystems; Table 3).

**Table 3:** TaqMan primer and probe sequences for COX2 and ACTB used in quantitative polymerase chain reaction quantification of mitochondrial DNA content in longissimus muscle.

Gene ID	Gene	Primer and probe sequence (5ʹ–3ʹ)	Manufacturer
**3283880**	*COX2*		Thermo Fisher
	Forward	6FAM-TCGTCCCGTCCAGGCTTA-MGBNFQ	
	Reverse	6FAM-GTGGTTTGACCCGCAAATTT-MGBNFQ	
	Probe	6FAM-ATTACGGTCAATGCTC-MGBNFQ	
**280979**	*ACTB*		
	Forward	6FAM-TCACGGAGCGTGGCTACAG-MGBNFQ	
	Reverse	6FAM-TTGATGTCACGGACGATTTCC-MGBNFQ	
	Probe	6FAM-CACCACCACGGCCGA-MGBNFQ	

Each reaction (10 µL) contained 10 ng of template DNA and was performed in duplicate on a QuantStudio™ 6 Pro Real-Time PCR System (Applied Biosystems; Waltham, MA, United States). Thermal cycling conditions included an initial denaturation at 95 °C for 20 s, followed by 35 cycles of 95 °C for 1 s and 60 °C for 20 s, with fluorescence acquisition at the end of each extension step. The relative mtDNA content was calculated using the 2⁻ΔCt method, where ΔCt = Ct(*COX2*) − Ct(*ACTB*), such that higher values reflect greater mtDNA abundance relative to nuclear DNA.

#### Circulating IGF-1

Circulating IGF-1 concentrations were quantified in serum samples submitted to the MSU VDL using a commercially available chemiluminescent immunoassay (IDS-iSYS; Immunodiagnostic Systems, Boldon, Tyne & Wear, UK). Although this assay is validated for use in human serum, bovine and human IGF-1 share 100% amino acid sequence identity (Francis et al. 1986), supporting its applicability to bovine samples. Bovine-specific validation data are not available from the manufacturer, and results should be interpreted with this limitation in mind.

#### Health event management and sample inclusion

During the 63-d treatment period, one steer exhibited an elevated rectal temperature exceeding 39.7 °C. The animal was treated subcutaneously with Florfenicol and Flunixin Meglumine (Resflor Gold; Batch #3582101, Merck Animal Health, Rahway, NJ, United States). Treatment occurred outside the designated sampling periods and met the required withdrawal interval before harvest; therefore, all associated samples were retained for analysis.

### Statistical analysis

This study was conducted as a completely randomized design. Statistical analyses were performed in RStudio (version 2025.05.1 + 513; Posit Software, PBC, Boston, MA, United States) using the nlme, lmerTest, and emmeans packages. Graphical illustrations were generated using GraphPad Prism (version 10.4.2; Dotmatics, Boston, MA, United States). Significance was declared at *P *≤ 0.05, and tendencies were noted at 0.05 < *P *≤ 0.10. Denominator degrees of freedom for models fitted using nlme were determined using the inner–outer method implemented in that package, whereas the Kenward–Roger approximation was used for the circulating LUB treatment comparisons. Model assumptions were evaluated by visual inspection of normal Q–Q plots and plots of residuals against fitted values to assess residual normality and homogeneity of variance, respectively.

Individual-steer measurements of BW, ADG, carcass characteristics, meat quality, chemical composition, and texture were analyzed with pen as the experimental unit for treatment and individual steer as the observational unit (*n* = 4 pens per treatment; four steers per pen). Initial pen mean BW was included as a covariate. Data were analyzed using the following linear mixed-effects model: *Y_lps_* = *µ* + *T_l_* + *P_p_* + *D_0_* + *e_lps_*, where *Y_lps_* is the response measured on steer *s* in pen *p* receiving treatment *l*, *µ* is the overall mean, *T_l_* is the fixed effect of LUB supplementation (CON vs. LUB), *P_p_* is the random effect of pen nested within treatment, *D_0_* is the initial pen mean BW as a covariate, and *e_lps_* is the residual error. DMI and G:F were analyzed using pen as the experimental unit (*n* = 4 pens per treatment), with treatment as a fixed effect and initial pen mean BW as a covariate. Pairwise comparisons between treatments were conducted using Tukey’s adjustment.

Circulating LUB concentrations at d 28 and d 56 were analyzed separately using the following linear mixed-effects model: *Y_lps_* = *µ* + *T_l_* + *P_p_* + *e_lps_*, where *Y_lps_* is the circulating LUB concentration measured in steer *s* from pen *p* receiving treatment *l*, *µ* is the overall mean, *T_l_* is the fixed effect of LUB supplementation, *P_p_* is the random effect of pen nested within treatment, and *e_lps_* is the residual error. Pen was considered the experimental unit for treatment, and individual steer was considered the observational unit. The models were fitted using restricted maximum likelihood REML). Because all day 0 concentrations were recorded as zero, they provided no variation for covariate adjustment and were not included as covariates or response observations. Treatment comparisons at day 28 and day 56 were adjusted collectively using the Šidák method.

Serum biochemical parameters and circulating IGF-1 concentrations measured on d 28 and 56 were analyzed using linear mixed-effects models for repeated measures. Treatment, day, and their interaction were included as fixed effects, and the corresponding d 0 value was included as a covariate. The model was expressed as *Y_lmps_* = *µ* + *T_l_* + *D_m_* + *T_l_D_m_* + *B_0_* + *P_p_* + *S_s(p)_* + *e_lmps_*, where *µ* is the overall mean, *T_l_* is the fixed effect of LUB supplementation, *D_m_* is the fixed effect of day, *T_l_D_m_* is the treatment × day interaction, *B_0_* is the corresponding d 0 value included as a covariate, *P_p_* is the random effect of pen, *S_s(p)_* is the random effect of steer nested within pen, and *e_lmps_* is the residual error. A compound symmetry covariance structure was used to account for repeated measurements within steers, and models were fitted using REML. Pairwise comparisons among treatment-by-day least-squares means were performed using the Šidák adjustment. Day 0 values were used only as covariates and were not included as response observations in the repeated-measures analysis. Day 0 serum IGF-1 concentrations are presented descriptively in Table 12.

Changes from baseline in gene expression were analyzed with pen as the experimental unit for treatment and individual steer as the observational unit. For each steer, the change from the d 0 baseline was calculated for d 28 and 56 (Δ = day x − day 0). These delta values were analyzed using a linear mixed-effects model for repeated measures: Δ*Y_lmps_* = *µ* + *T_l_* + *D_m_* + *T_l_D_m_* + *P_p_* + *S_s(p)_* + *e_lmps_*, where *µ* is the overall mean, *T_l_* is the fixed effect of LUB supplementation, *D_m_* is the fixed effect of day, *T_l_D_m_* is the treatment × day interaction, *P_p_* is the random effect of pen, *S_s(p)_* is the random effect of steer nested within pen, and *e_lmps_* is the residual error. A first-order autoregressive covariance structure was used to account for repeated measurements within steers, and the models were fitted using REML. Because the response variable was the change score (Δ), d 0 baseline was inherently accounted for, and no additional baseline covariate was included. Post hoc pairwise comparisons were conducted using the Šidák adjustment when the treatment × day interaction was significant. Samples with undetermined ARB3 Ct values (*n* = 13 of 32 samples; 40.6%) were excluded from statistical analysis of ARB3 expression. A sensitivity analysis was conducted using only samples with detectable ARB3 expression (*n* = 19) to confirm that conclusions regarding treatment effects were not influenced by exclusion of undetectable samples. As a sensitivity analysis, all molecular end points were additionally analyzed with individual steer as the experimental unit; conclusions were consistent with the pen-based models for the majority of endpoints, and the pen-based results are reported throughout. A comparison of treatment *P*-values obtained under the two approaches for LM protein abundance is provided in Table S3.

Changes in protein abundance from baseline were evaluated with pen as the experimental unit for treatment and individual steer as the observational unit. For each steer, the change from d 0 to d 56 was calculated (Δ = day 56 − day 0). These delta values were analyzed using the following linear mixed-effects model: Δ*Y_lps_* = *µ* + *T_l_* + *P_p_* + *e_lps_*, where *Y_lps_* is the response measured on steer *s* in pen *p* receiving treatment *l*, *µ* is the overall mean, *T_l_* is the fixed effect of LUB supplementation, *P_p_* is the random effect of pen, and *e_lps_* is the residual error. The models were fitted using REML. Because the response variable was the change score (Δ), the d 0 baseline was inherently accounted for, and no additional baseline covariate was included. Differences between treatments were evaluated using the Šidák adjustment.

The mtDNA: gDNA ratio at d 56 was analyzed using a linear mixed-effects analysis of covariance model, with pen as the experimental unit for treatment and individual steer as the observational unit. The corresponding d 0 mtDNA: gDNA ratio for each steer was included as a covariate. Data were analyzed using the following model: *Y_lps_* = *µ* + *T_l_* + *B*_0_ + *P_p_* + *e_lps_*, where *Y_lps_* is the d 56 mtDNA: gDNA ratio measured on steer *s* in pen *p* receiving treatment *l*, *B*_0_ is the corresponding d 0 value included as a covariate, and the remaining terms are as defined above. The model was fitted using REML. Day 0 values were used only as covariates and were not included as response observations. Differences between treatments were evaluated using the Šidák adjustment.

## Results

### Growth performance

**Figure 1:**
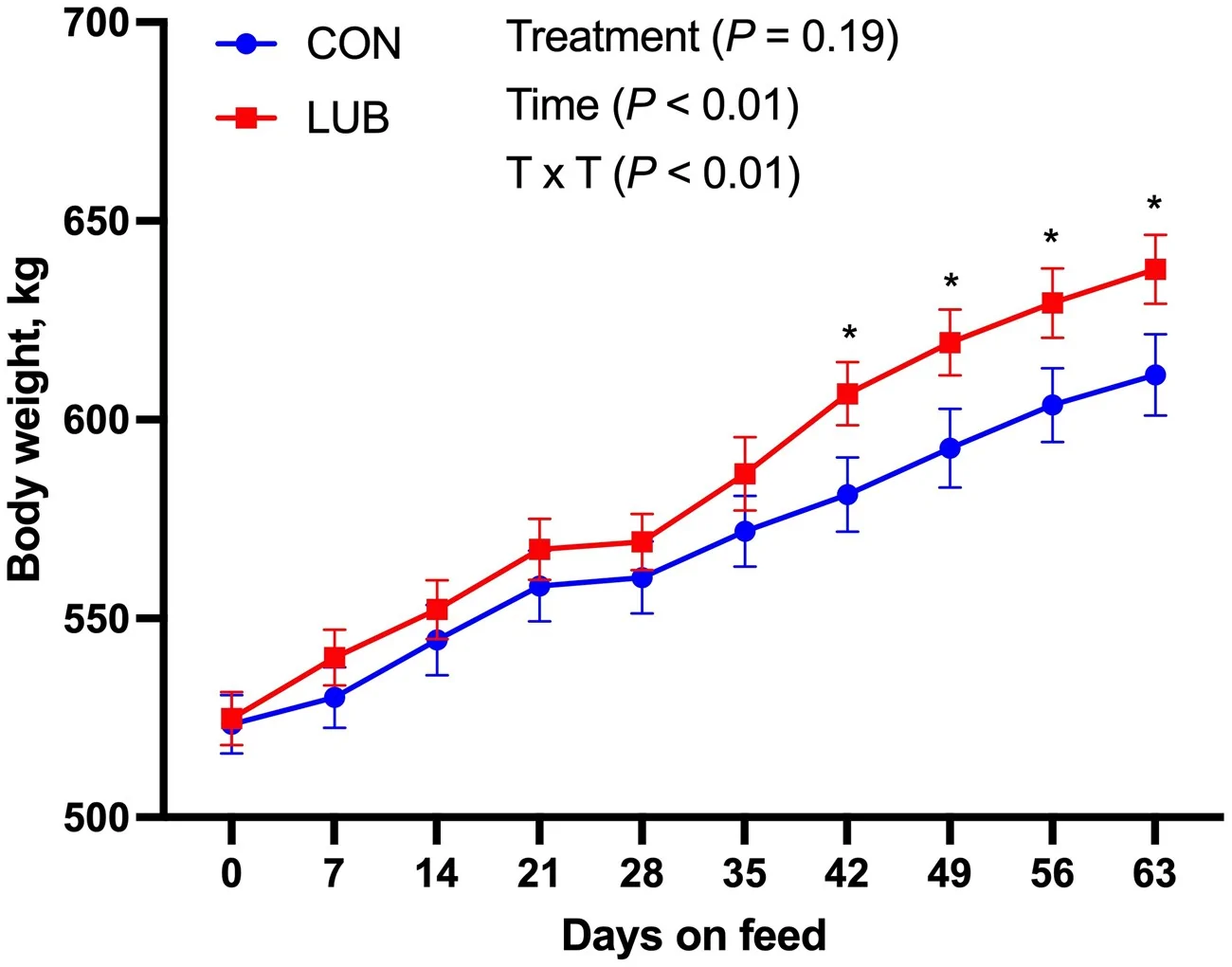
Effects of lubabegron supplementation on body weight over the 63-d finishing period. Body weights were measured weekly to assess treatment effects over time. Data are presented as means ± SEM for control (CON; blue) and lubabegron-treated (LUB; red) steers. The effects of time and the treatment × time interaction were significant (*P* < 0.01), whereas the overall treatment effect was not significant (*P* = 0.19). LUB-treated steers had greater BW than CON steers at d 42, 49, 56, and 63. An asterisk indicates a significant difference between treatments within a sampling day (*P* < 0.05).

Feeding LUB for 63 d significantly enhanced growth performance in finishing beef steers (Table 4, Figure 1). Steers supplemented with LUB had a higher final BW compared to controls (637.0 kg vs. 612.2 kg; *P* < 0.01). ADG was also improved in the LUB group (1.79 kg/d) relative to CON (1.40 kg/d; *P* < 0.01). Additionally, LUB-fed steers showed increased DMI (*P* < 0.01), consuming an average of 10.20 kg/day compared to 9.32 kg/day in the CON group. Feed efficiency, measured by G:F, was significantly improved in the LUB steers (*P* < 0.01).

**Table 4:** Growth performance of finishing beef steers supplemented with lubabegron (LUB) or fed a control diet (CON) for 63 d.

Item	Treatments^1^		SEM^2^	*P*-value
	CON	LUB		
**Number of pens**	4	4		
**Initial body weight (BW), kg**	523.40	524.82	15.19	0.95
**Final BW, kg**	612.16	636.99	4.01	<0.01
**ADG, kg/day**	1.40	1.79	0.06	<0.01
**DMI, kg/day**	9.32	10.20	0.13	<0.01
**G:F**	0.14	0.17	0.00	<0.01

1 CON = basal diet without lubabegron supplementation; LUB = basal diet supplemented with lubabegron at 3.5 mg/kg DM. Data are least squares means with pen as the experimental unit (*n* = 4 pens per treatment; four steers per pen). Initial BW was included as a covariate.

2 Pooled standard error of the mean.

### Circulating LUB

All day 0 serum LUB concentrations were recorded as zero and are presented descriptively. At day 28 and day 56, serum LUB concentrations remained zero in CON steers but averaged 1.80 and 1.60 nM in LUB-supplemented steers, respectively (Šidák-adjusted *P* < 0.01 for both sampling days; Table 5).

**Table 5:** Serum lubabegron levels in LUB-supplemented beef finishing steers.

Day	Treatments	Serum LUB, nM	**SEM** ^1^	***P*-value** ^2^
**0^3^**	CON	0	–	–
	LUB	0		
**28 **	CON	0	0.20	<0.01
	LUB	1.80		
**56 **	CON	0	0.15	<0.01
	LUB	1.60		

1 Pooled standard error of the mean for each sampling day.

2 *P*-values for day 28 and day 56 were obtained from separate pen-based linear mixed-effects models and adjusted collectively using the Šidák method.

3 All d 0 values were recorded as zero, presented descriptively, and excluded from statistical analysis.

### Serum chemistry profile

Treatment × time interactions were detected for urea nitrogen, potassium, and ALP (*P* = 0.04, *P* = 0.03, and *P* = 0.02, respectively; Table 6). Šidák-adjusted pairwise comparisons detected no differences among the treatment × time least-squares means for urea nitrogen or potassium (*P* > 0.05). For ALP, activity in LUB steers was greater at d 28 than at d 56, whereas the remaining treatment × time least-squares means did not differ (Table 6). Tendencies for treatment × time interactions were observed for the Na:K ratio and total and indirect bilirubin (*P* = 0.07 and *P* = 0.09, respectively).

**Table 6:** Effects of lubabegron supplementation on serum biochemical parameters in finishing steers.

Serum parameters		Treatment^1^		SEM	***P*-value** ^2^		
		CON	LUB		Treatment	Time	**T** × **T**
**Urea nitrogen, mg/dL**	Day 28	11.33	11.67	0.65	0.16	0.61	0.04
	Day 56	12.14	11.17				
**Creatinine, mg/dL**	Day 28	1.10	1.21	0.02	0.01	0.85	0.35
	Day 56	1.09	1.23				
**Sodium, mmol/L**	Day 28	142.03	141.85	0.25	0.42	0.04	0.55
	Day 56	141.46	141.54				
**Potassium, mmol/L**	Day 28	3.82	3.80	0.06	0.16	0.42	0.03
	Day 56	3.75	3.94				
**Chloride, mmol/L**	Day 28	98.21	98.73	0.33	0.75	0.47	0.81
	Day 56	98.33	98.98				
**TCO2, mmol/L**	Day 28	30.72	31.91	0.50	0.08	0.19	0.48
	Day 56	31.53	32.16				
**NaK ratio**	Day 28	37.39	37.55	0.64	0.17	0.18	0.07
	Day 56	37.64	36.05				
**Anion gap, mmol/L**	Day 28	16.85	15.15	0.49	0.03	0.01	0.46
	Day 56	15.54	14.40				
**Osmolarity Calc, mmol/L**	Day 28	293.34	293.41	0.70	0.86	0.04	0.95
	Day 56	292.40	292.41				
**Ca, mg/dL**	Day 28	9.51	9.52	0.07	0.18	0.65	0.24
	Day 56	9.40	9.57				
**P, mg/dL**	Day 28	6.55	6.53	0.16	0.63	0.98	0.68
	Day 56	6.50	6.58				
**Mg, mg/dL**	Day 28	2.05	2.10	0.04	0.79	0.79	0.12
	Day 56	2.08	2.06				
**Total protein, g/dL**	Day 28	7.47	7.67	0.07	<0.01	0.25	0.92
	Day 56	7.40	7.60				
**Albumin, g/dL**	Day 28	3.63	3.62	0.04	0.19	0.20	0.41
	Day 56	3.64	3.67				
**Globulin calc, g/dL**	Day 28	3.86	4.02	0.08	0.01	0.07	0.78
	Day 56	3.78	3.91				
**Glucose, mg/dL**	Day 28	97.21	96.60	1.46	0.27	0.05	0.13
	Day 56	93.09	96.04				
**Total bili, mg/dL**	Day 28	0.14	0.16	0.01	0.01	0.09	0.09
	Day 56	0.17	0.16				
**Indirect bili, mg/dL**	Day 28	0.14	0.16	0.01	0.01	0.09	0.09
	Day 56	0.17	0.16				
**ALP, U/L**	Day 28	147.24^ab^	173.69^a^	7.07	0.03	0.04	0.02
	Day 56	149.68^ab^	148.44^b^				
**GGT, U/L**	Day 28	24.10	23.28	1.73	0.43	0.72	0.83
	Day 56	24.22	23.78				
**AST, U/L**	Day 28	72.12	74.63	3.92	0.69	0.62	0.97
	Day 56	73.18	75.88				
**CK, U/L**	Day 28	255.40	286.16	67.30	0.43	0.43	0.42
	Day 56	177.02	287.48				
**Chol, mg/dL**	Day 28	135.45	144.80	5.21	0.02	0.36	0.66
	Day 56	133.70	139.86				

T × T = treatment × time interaction.

1 Dietary inclusion of LUB or CON; time = sampling day (d 28 or 56).

2 Values are baseline-adjusted least-squares means. The corresponding d 0 value was included as a covariate. *P*-values represent the fixed effects of treatment, time, and their interaction from a repeated-measures linear mixed-effects model with pen and steer nested within pen as random effects. Significant treatment × time interactions were detected for serum urea nitrogen and potassium; however, Šidák-adjusted pairwise comparisons detected no differences among the corresponding treatment × time least-squares means (*P* > 0.05)

a,b Within an analyte, treatment × time least-squares means with different superscripts differ (*P* ≤ 0.05; Šidák-adjusted). Superscripts are shown only when at least one pairwise difference was detected; the absence of superscripts indicates that no significant pairwise differences were detected.

Among variables without a significant treatment × time interaction, creatinine concentrations were greater in LUB steers than in CON steers (*P* = 0.01). Total protein (*P* < 0.01), globulin (*P* = 0.01), total and indirect bilirubin (*P* = 0.01), and cholesterol (*P* = 0.02) were also greater in LUB steers, whereas the anion gap was lower (*P* = 0.03). TCO₂ tended to be greater in LUB steers (*P* = 0.08).

### Carcass traits

Feeding LUB for 63 d significantly increased HCW compared to the control group (382.26 kg vs. 363.73 kg; *P* < 0.01), resulting in an average increase of approximately 18.5 kg per steer (Table 7). Despite this increase, dressing percentage did not differ between treatments (59.99% vs. 59.42%; *P* = 0.25). Dressing percentage was calculated from unshrunk final BW; application of a standard 4% pencil shrink would raise the estimate to approximately 62.5% (LUB) and 61.9% (CON), within the range reported in comparable finishing trials.

**Table 7:** Carcass characteristics and meat quality parameters of finishing steers fed with or without Lubabegron for 63 d.

**Item** ^1^	Treatments		SEM	*P*-value
	CON	LUB		
**Hot carcass weight, kg**	363.73	382.26	2.35	<0.01
**Dressed yield, %**	59.42	59.99	0.32	0.25
**Ribeye area, cm** ^2^	89.09	92.28	1.86	0.27
**12th-rib fat thickness, cm**	1.33	1.40	0.10	0.66
**KPH, %**	1.42	1.40	0.05	0.73
**Calculated yield grade**	2.73	2.78	0.15	0.82
**Marbling score**	473.66	438.84	22.82	0.32
**Ultimate pH**	5.49	5.43	0.05	0.43
**Lean color**				
L*	32.02	33.48	0.79	0.24
a*	19.50	18.41	0.50	0.18
b*	13.03	12.28	0.43	0.26

1 Marbling score is based on the United States Department of Agriculture (USDA) scale, where 400 = Slight00 and 500 = Small00; lean color values are based on the CIE Lab* color space: L* = lightness, a* = redness, b* = yellowness.

REA was numerically greater in the LUB group (92.28 cm^2^) than in the control group (89.09 cm^2^), but this difference was not statistically significant (*P* = 0.27). Similarly, 12th-rib fat thickness did not differ (1.40 cm vs. 1.33 cm; *P* = 0.66), nor KPH fat percentage (1.40% vs. 1.42%; *P* = 0.73). Calculated USDA yield grade was also unaffected by treatment (2.78 vs. 2.73; *P* = 0.82).

Marbling score did not differ between treatments (LUB 438.84 vs. CON 473.66; *P* = 0.32). Additionally, no treatment effect was observed on ultimate muscle pH (5.43 vs. 5.49; *P* = 0.43), indicating similar extents of postmortem glycolysis across treatment groups.

Lean color measurements, including L* (lightness), a* (redness), and b* (yellowness), were not significantly affected by LUB supplementation. Lean color did not differ between LUB and CON: L* (33.48 vs. 32.02; *P* = 0.24), a* (18.41 vs. 19.50; *P* = 0.18), and b* (12.28 vs. 13.03; *P* = 0.26).

### Proximate composition and texture analysis

LUB supplementation influenced both the chemical composition and tenderness of the LM in finishing steers (Table 8). LUB-fed steers had significantly higher crude protein content compared to controls (23.60% vs. 22.47%; *P* < 0.01). There was also a trend toward lower crude fat content in LUB-fed steers (5.04% vs. 6.39%; *P* = 0.051). Moisture content did not differ between treatments (71.61% vs. 71.12%; *P* = 0.31).

**Table 8:** Chemical composition and texture analysis of LM of steers fed LUB for 63 d.

Items	Treatments		SEM	*P*-value
	CON	LUB		
**Compositional analysis**				
**Moisture, %**	71.12	71.61	0.31	0.31
**Protein, %**	22.47	23.60	0.17	<0.01
**Fat, %**	6.39	5.04	0.39	0.051
**Texture analysis**				
**Cooking loss, %^1^**				
**Day 2**	13.70	11.86	1.31	0.16
**Day 14**	14.35	14.84	1.31	0.71
**WBSF, kg**^2^				
**Day 2**	3.67	4.36	0.22	0.03
**Day 14**	2.76	3.74	0.22	<0.01

1 A higher value indicates reduced tenderness.

^2^Cooking loss and chemical composition were measured on LM samples aged for 2 or 14 d postmortem.

For texture attributes, cooking loss was not significantly different between treatments at either postmortem aging time point. At d 2, cooking loss averaged 11.86% in LUB-fed and 13.70% in control steers (*P* = 0.16). At d 14, values were similar (14.84% vs. 14.35%; *P* = 0.71).

However, WBSF was affected by treatment. At d 2, LUB-fed steers had higher WBSF values (4.36 kg vs. 3.67 kg; *P* = 0.03), indicating reduced tenderness. This difference became more pronounced by d 14 postmortem, with LUB-fed steers showing greater WBSF values (3.74 kg vs. 2.76 kg; *P* < 0.01), suggesting less improvement in tenderness with aging.

### RNA sequencing

Across samples, 51–82 million raw reads were generated (average 69 million) with >99% passing quality control and trimming. Of trimmed reads, 94.7–96.4% aligned to the reference genome, with concordant, uniquely mapped reads averaging 55.9 million per sample (81.3% of total mapped reads).

**Figure 2:**
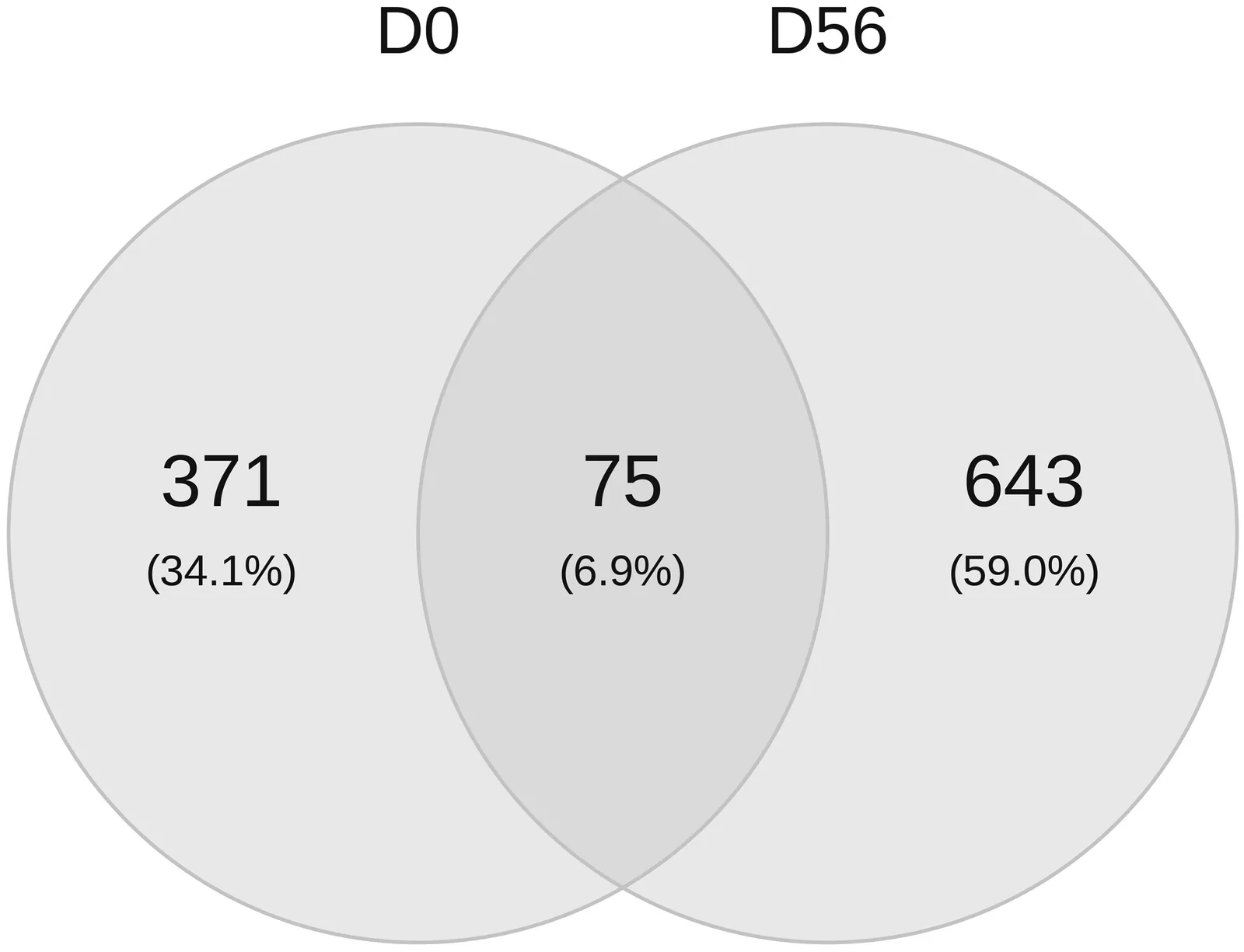
Differentially expressed genes (DEGs) in the LM of cattle treated with LUB for 56 d compared to baseline (D0). A total 643 of DEGs were identified at d 56 (FDR < 0.1). Gene expression differences were determined by RNA sequencing and analyzed using a false discovery rate (FDR) threshold of 10%.

At d 56, a total of 643 DEGs were identified between LUB-treated and control cattle (FDR < 0.1). Of these, 523 genes were upregulated, and 120 genes were downregulated in the treated group (Figure 2).

Upregulated genes included several associated with myogenesis, energy metabolism, and stress response, such as *MSTN, RRAGD, FZD7, MYF6, SOX6, MEF2A, PLPP7, MAP2K2, KLF5, MYH1, SRF, MAPK6, ZBTB7C, TMEM64, PRKAB2, PFKM, EEF2K, PKM, NCOA1, ZBTB16, HSPB2, HSP90AA1, ATF4*, and *SHISA2*. The 25 most significantly upregulated genes (based on adjusted *P*-value) are listed in Table 9. Among upregulated genes, myostatin *(MSTN)*, a well-established negative regulator of skeletal muscle mass, was identified as a DEG at d 56 (Table 9).

**Table 9:** Differentially expressed (upregulated) genes related to muscle growth, metabolism, and stress response in steers supplemented with lubabegron.

Gene symbol^1^	Description	log2 FC	FDR
***WWP1***	WW domain containing E3 ubiquitin protein ligase 1	0.84	1.09E-09
***ATF4***	Activating transcription factor 4	0.62	1.65E-07
***ZBTB7C***	Zinc finger and BTB domain containing 7C	0.77	3.45E-06
***SHISA2***	SHISA family member 2	1.20	9.53E-06
***HSPB2***	Heat shock protein family B (small) member 2	0.45	1.66E-05
***MSTN***	Myostatin	1.26	1.70E-05
***RRAGD***	Ras related GTP binding D	0.58	2.49E-04
***FZD7***	Frizzled class receptor 7	0.72	2.80E-04
***MYF6***	Myogenic factor 6	0.68	3.90E-04
***TMEM64***	Transmembrane protein 64	0.64	5.62E-04
***SOX6***	SRY-box transcription factor 6	0.80	6.66E-04
***MEF2A***	Myocyte enhancer factor 2A	0.50	8.19E-04
***PLPP7***	Phospholipid phosphatase 7 (inactive)	0.61	9.74E-04
***PRKAB2***	Protein kinase AMP-activated non-catalytic subunit beta 2	0.50	5.01E-03
***PFKM***	Phosphofructokinase, muscle	0.59	5.61E-03
***EEF2K***	Eukaryotic elongation factor 2 kinase	0.49	1.35E-02
***MAP2K2***	Mitogen-activated protein kinase kinase 2	0.33	1.35E-02
***HSP90AA1***	Heat shock protein 90 alpha family class A member 1	0.32	1.45E-02
***PKM***	Pyruvate kinase M1/2	0.59	1.60E-02
***NCOA1***	Nuclear receptor coactivator 1	0.36	1.76E-02
***KLF5***	Kruppel like factor 5	0.68	2.20E-02
***MYH1***	Myosin heavy chain 1	0.75	3.08E-02
***SRF***	Serum response factor	0.46	3.50E-02
***MAPK6***	Mitogen-activated protein kinase 6	0.53	4.08E-02
***ZBTB16***	Zinc finger and BTB domain containing 16	0.64	4.15E-02

1 Gene symbols and descriptions are based on NCBI gene annotations. Log₂ FC represents the log₂ fold change in gene expression relative to control or baseline conditions. Adjusted *P*-values were calculated using a false discovery rate (FDR) correction to account for multiple testing.

Conversely, genes involved in transcriptional regulation, metabolic signaling, and lipid storage regulation, such as *ZNF750*, *NR4A2*, *HES1*, *TRIB1*, *LPL*, *FABP3*, and *PLIN5*, were significantly downregulated. The 25 most significantly downregulated genes are also shown in Table 10, with *ZNF750* (log₂FC = −1.64, FDR = 1.4 × 10⁻¹^5^) and *RASL12* (log₂FC = −0.84, FDR = 1.4 × 10^−7^) among the most significantly downregulated genes.

**Table 10:** Differentially expressed (downregulated) genes related to muscle growth, metabolism, and stress response in steers supplemented with Lubabegron.

Gene symbol^1^	Description	log2 FC	FDR
***ZNF750***	Zinc finger protein 750	−1.64	1.39E-15
***RASL12***	RAS like family 12	−0.84	1.38E-07
***HES1***	HES family BHLH transcription factor 1	−2.01	7.08E-06
***NR4A2***	Nuclear receptor subfamily 4 group A member 2	−2.99	4.13E-05
***CRHR1***	Corticotropin releasing hormone receptor 1	−1.21	1.46E-04
***RORC***	RAR related orphan receptor C	−1.16	1.54E-04
***ARRDC3***	Arrestin domain containing 3	−0.78	2.20E-04
***IFFO2***	Intermediate filament family orphan 2	−0.66	3.37E-04
***WDR86***	WD repeat domain 86	−2.31	5.62E-04
***TMEM100***	Transmembrane protein 100	−0.97	7.81E-04
***SLC1A3***	Solute carrier family 1 member 3	−0.59	8.19E-04
***TRIB1***	Tribbles pseudokinase 1	−0.94	8.19E-04
***LPL***	Lipoprotein lipase	−1.21	1.85E-03
***RELT***	RELT TNF receptor	−0.97	3.10E-03
***GADD45A***	Growth arrest and DNA damage inducible alpha	−0.96	3.82E-03
***TCIM***	Transcriptional and immune response regulator	−0.81	3.82E-03
***ARRDC4***	Arrestin domain containing 4	−0.98	7.67E-03
***FABP3***	Fatty acid binding protein 3	−0.74	8.48E-03
***COX14***	Cytochrome c oxidase assembly factor COX14	−0.70	1.08E-02
***METRNL***	Meteorin like, glial cell differentiation regulator	−0.84	1.31E-02
***GPAM***	Glycerol-3-phosphate acyltransferase, mitochondrial	−0.45	1.35E-02
***SMPX***	Small muscle protein X-linked	−0.70	1.54E-02
***RDH13***	Retinol dehydrogenase 13	−0.65	1.69E-02
***ASPN***	Asporin	−0.87	1.76E-02
***PLIN5***	Perilipin 5	−0.91	1.76E-02

1 Gene symbols and descriptions are based on NCBI gene annotations. Log₂ FC represents the log₂ fold change in gene expression relative to control or baseline conditions. Adjusted *P*-values were calculated using a false discovery rate (FDR) correction to account for multiple testing.

**Figure 3:**
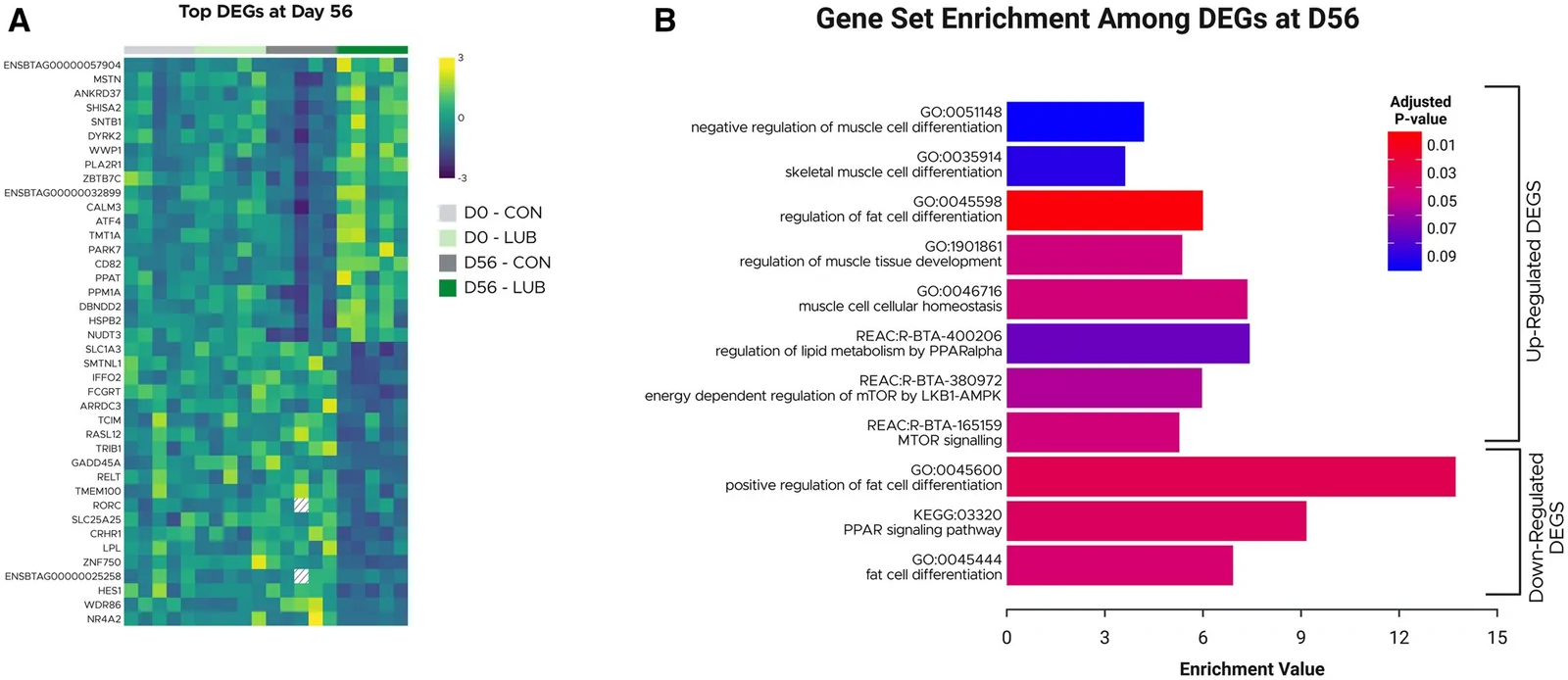
Transcriptomic changes in longissimus muscle of steers supplemented with Lubabegron (LUB) for 56 d. (A) Heatmap showing the top differentially expressed genes (DEGs) (false discovery rate [FDR] < 0.01) identified by RNA sequencing at d 56. Gene expression values are scaled (*Z*-score) and log₂-transformed. Columns represent individual samples across treatment and time points: Day 0 control (D0-control [CON]), Day 0 LUB (D0-LUB), Day 56 control (D56-CON), and Day 56 LUB (D56-LUB). (B) Gene set enrichment analysis (GSEA) of DEGs at d 56. Bars represent enriched Gene Ontology (GO) terms and Reactome/KEGG pathways related to muscle development, lipid metabolism, and energy regulation. Enrichment value corresponds to the number of DEGs associated with each pathway. Color gradient reflects adjusted *P*-values, with red indicating higher statistical significance. Pathways are grouped by whether they were enriched among up-regulated or down-regulated DEGs in the LUB-treated group. Figures created in https://BioRender.com

DEGs were enriched for biological processes related to muscle tissue development, fat cell differentiation, and pathways such as mTOR signaling and PPAR signaling (Figure 3).

### Gene expression (RT-qPCR)

Expression of genes related to β-adrenergic signaling, myogenesis, fiber type composition, and adipogenesis was evaluated at d 28 and 56 relative to d 0 (Table 11).

**Table 11:** Skeletal muscle gene expression changes from baseline in beef steers supplemented with lubabegron or fed a control diet for 63 d.

Genes		Treatment		SEM	***P*-value** ^1^		
		CON	LUB		Treatment	Time	T x T
***ADRB1***	Day 28	−0.49	−0.57	0.27	0.65	0.68	0.17
	Day 56	−0.36	−0.64				
***ADRB2***	Day 28	−0.22	−0.21	0.16	0.59	0.43	0.45
	Day 56	−0.41	−0.21				
***ADRB3* ^2^**	Day 28	−0.42	−0.02	0.30	0.79	0.05	0.19
	Day 56	−0.52	−0.46				
***CREB***	Day 28	−0.17	−0.24	0.10	0.78	<0.01	0.49
	Day 56	−0.04	−0.05				
***IGF1***	Day 28	−0.46	−0.12	0.17	0.11	0.04	0.64
	Day 56	−0.23	0.23				
***MSTN***	Day 28	−0.12	−0.24	0.19	0.90	<0.01	0.11
	Day 56	0.07	0.26				
***MYF5***	Day 28	−0.43	0.01	0.20	0.07	0.01	0.58
	Day 56	−0.04	0.57				
***MYOD1***	Day 28	0.00	0.33	0.16	0.55	0.02	0.13
	Day 56	−0.15	−0.26				
***MYOG***	Day 28	−0.29^b^	1.00^a^	0.18	0.01	0.16	0.03
	Day 56	−0.13^b^	0.32^ab^				
***MYH7***	Day 28	0.05	0.00	0.18	0.14	0.66	0.06
	Day 56	0.42	−0.23				
***MYH2***	Day 28	−0.50	0.45	0.16	0.27	0.10	0.65
	Day 56	0.03	1.34				
***MYH1***	Day 28	−0.80	−0.09	0.75	0.26	0.01	0.32
	Day 56	0.22	2.08				
***C/EBPα***	Day 28	−0.36	−0.47	0.22	0.57	0.33	0.25
	Day 56	−0.23	−0.48				
***PPARα***	Day 28	0.03	−0.51	0.14	0.03	0.05	0.50
	Day 56	0.17	−0.23				
***PPARγ***	Day 28	−0.19	−0.14	0.16	0.58	<0.01	0.55
	Day 56	0.17	0.39				
***PGC-1α***	Day 28	0.26^a^	−0.23^b^	0.10	0.05	0.26	0.03
	Day 56	−0.07^ab^	−0.12^ab^				
***FABP4***	Day 28	−0.81	0.25	0.85	0.20	<0.05	0.39
	Day 56	0.06	2.34				
***ZNF423***	Day 28	−0.12	−0.14	0.06	0.88	0.30	0.94
	Day 56	−0.07	−0.08				
***TAB1***	Day 28	−0.20	−0.11	0.08	0.84	<0.01	0.27
	Day 56	0.10	0.06				
***COL1A1***	Day 28	−0.86	0.42	0.56	0.17	0.18	0.77
	Day 56	−0.96	0.26				

MYF5, MYOD1, MYOG = myogenic regulatory factors; MYH1, MYH2, MYH7 = myosin heavy chain isoforms (IIx, IIa, I).

1 Values represent log₂ fold changes in gene expression relative to baseline (d 0). *P*-values reflect the fixed effects of treatment (Trt), time (d 28 vs. d 56), and their interaction (T × T) from repeated-measures linear mixed-effects model.

2 ADRB3 transcript was undetectable in 13 of 32 samples (40.625%; Ct range of detectable samples: 36.12–39.85). Statistical analysis of ADRB3 was performed using only samples with detectable expression (*n* = 19). Treatment effect was not significant in either the full dataset or the detectable-only subset.

a,b Means within a row with different superscripts differ significantly (*P* < 0.05). Superscript letters are not shown for MYH7, as pairwise comparisons among treatment × time means were not significant (*P* > 0.05, Šidák-adjusted), despite significant or trending effects observed in the model.

**Table 12:** Effects of dietary lubabegron supplementation on serum IGF-1 concentrations in finishing steers.

		Treatment^1^		SEM^2^	*P*-value^3^		
		CON	LUB		Treatment	Time	T x T
	Day 0	441.94	453.25	17.16	0.66	<0.01	<0.05
**IGF-1, ng/mL**	Day 28	444.47^a^	451.71^ab^				
	Day 56	397.97^bc^	358.09^c^				

1 Dietary inclusion of lubabegron (LUB) or control (CON).

2 Pooled standard error of the least-squares means.

3 *P*-values reflect the fixed effects of treatment (Trt), time (d 28 vs. 56), and their interaction (Trt × Time) from a linear mixed-effects model for repeated measures, with the corresponding d 0 IGF-1 concentration included as a covariate. Pen and steer nested within pen were included as random effects, and a compound symmetry covariance structure was used to account for repeated measurements within steers. The model was fitted using restricted maximum likelihood (REML). Day 0 values are presented descriptively and were not included as response observations.

a,b,c Means within different superscripts differ significantly (*P* < 0.05) based on Šidák-adjusted pairwise comparisons.

A treatment × time interaction was detected for *MYOG* (*P* = 0.03), where expression was increased in the LUB group at d 28 compared with CON. A similar interaction was observed for *PGC-1α* (*P* = 0.03), with expression reduced in the LUB group at d 28 relative to d 0, while expression increased in the CON group at the same timepoint.

A tendency for a main effect of treatment was detected for *MYF5* (*P* = 0.07), with numerically greater expression in LUB compared with CON averaged across timepoints. *IGF1* expression did not differ between treatments (*P* = 0.11) but increased over time (*P* = 0.04), with the numerically greatest values in LUB steers at d 56. No treatment × time interactions were observed for these genes (*P* > 0.10).

Among *MHC* isoforms, expression of *MYH1* increased over time regardless of treatment (*P* = 0.01), while *MYH2* tended to increase over time (*P* = 0.10). No treatment × time interactions were detected for either gene (*MYH1*, *P* = 0.32; *MYH2*, *P* = 0.65), and treatment effects were not significant (*P* > 0.10). *MYH7* mRNA expression did not differ by treatment (*P* = 0.14) or time (*P *= 0.66); however, a tendency for a treatment × time interaction was detected (*P *=  0.06).

No treatment effect or treatment × time interaction was detected for *MYOD1* (*P *= 0.55 and *P *= 0.13, respectively), although its expression decreased over time (*P *= 0.02). Similarly, *MSTN* and *CREB* did not differ between treatments (*P *= 0.90 and *P *= 0.78, respectively) and showed no treatment × time interactions (*P *= 0.11 and *P *= 0.49, respectively); however, the expression of both genes increased over time (*P *< 0.01).

*FABP4* mRNA expression did not differ between treatments (*P* = 0.20), and no treatment × time interaction was detected (*P* = 0.39). Expression of *PPARα* was lower in LUB-supplemented steers than in CON (*P* = 0.03), consistent with reduced transcriptional support for fatty acid oxidation and lipid handling. No treatment effects were detected for *PPARγ* (*P* = 0.58) or *C/EBPα* (*P *= 0.57), although *PPARγ* increased over time (*P* < 0.01).

No treatment effects were observed for *ARB1*, *ARB2*, or *ARB3* (*P *= 0.65, 0.59, and 0.79, respectively). *ARB3* transcript abundance was undetectable in 13 of 32 samples (40.6%), with detectable Ct values ranging from 36.12 to 39.85, indicating minimal and highly variable β-3 AR expression in LM tissue. Among samples with detectable expression, no treatment effect was observed, consistent with the full-dataset analysis. In contrast, *ARB1* and *ARB2* were consistently detectable across all samples (Ct range: 27.84–33.46 and 25.97–26.78, respectively), confirming adequate RNA quality and assay performance. No treatment effects were detected for *ZNF423*, *TAB1*, or *COL1A1* (*P* = 0.88, *P* = 0.84, and *P* = 0.17, respectively). However, *TAB1* expression increased over time (*P* < 0.01), whereas no time effects were detected for *ZNF423* or *COL1A1*.

### Anabolic and energy-sensing signaling pathways

**Figure 4:**
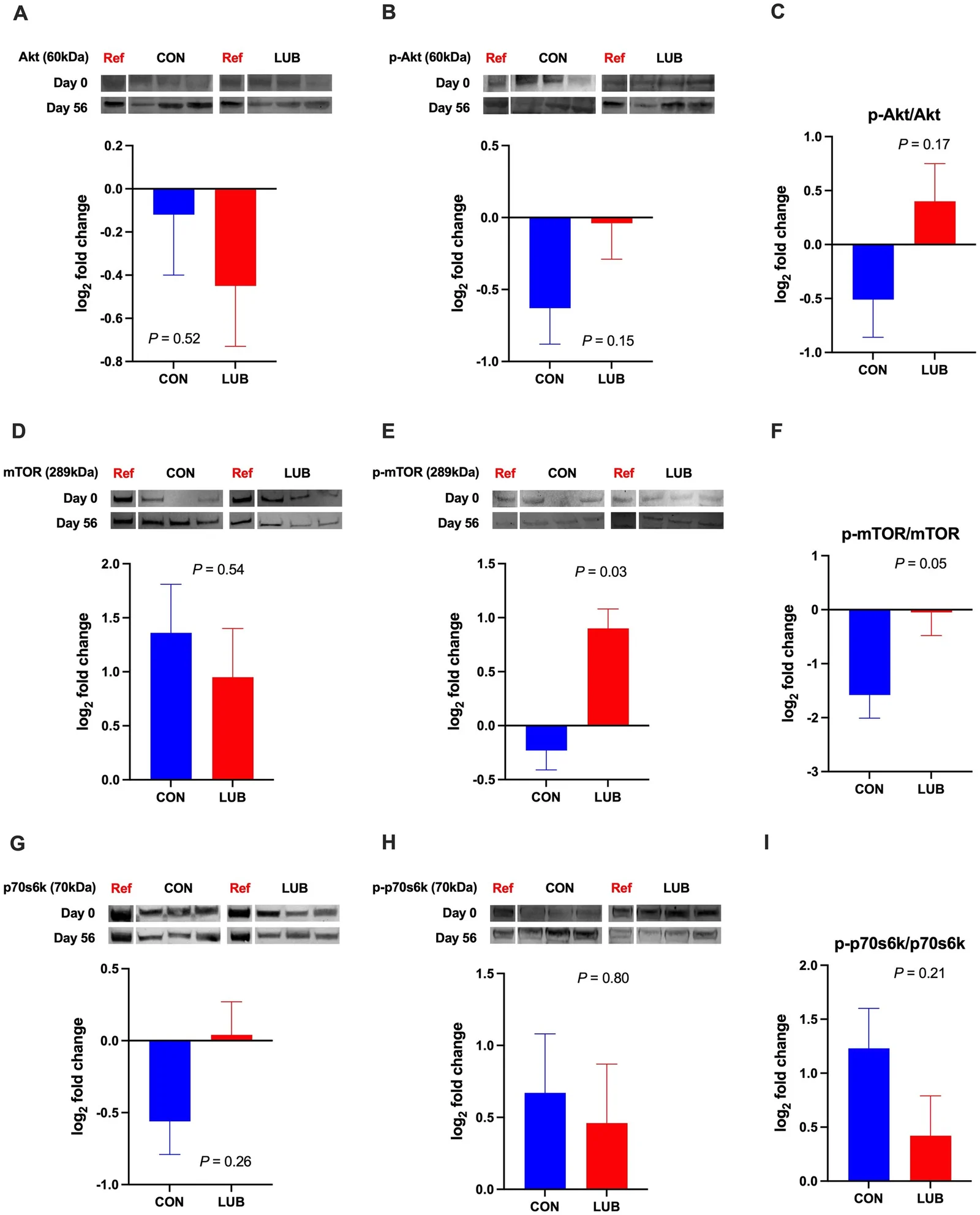

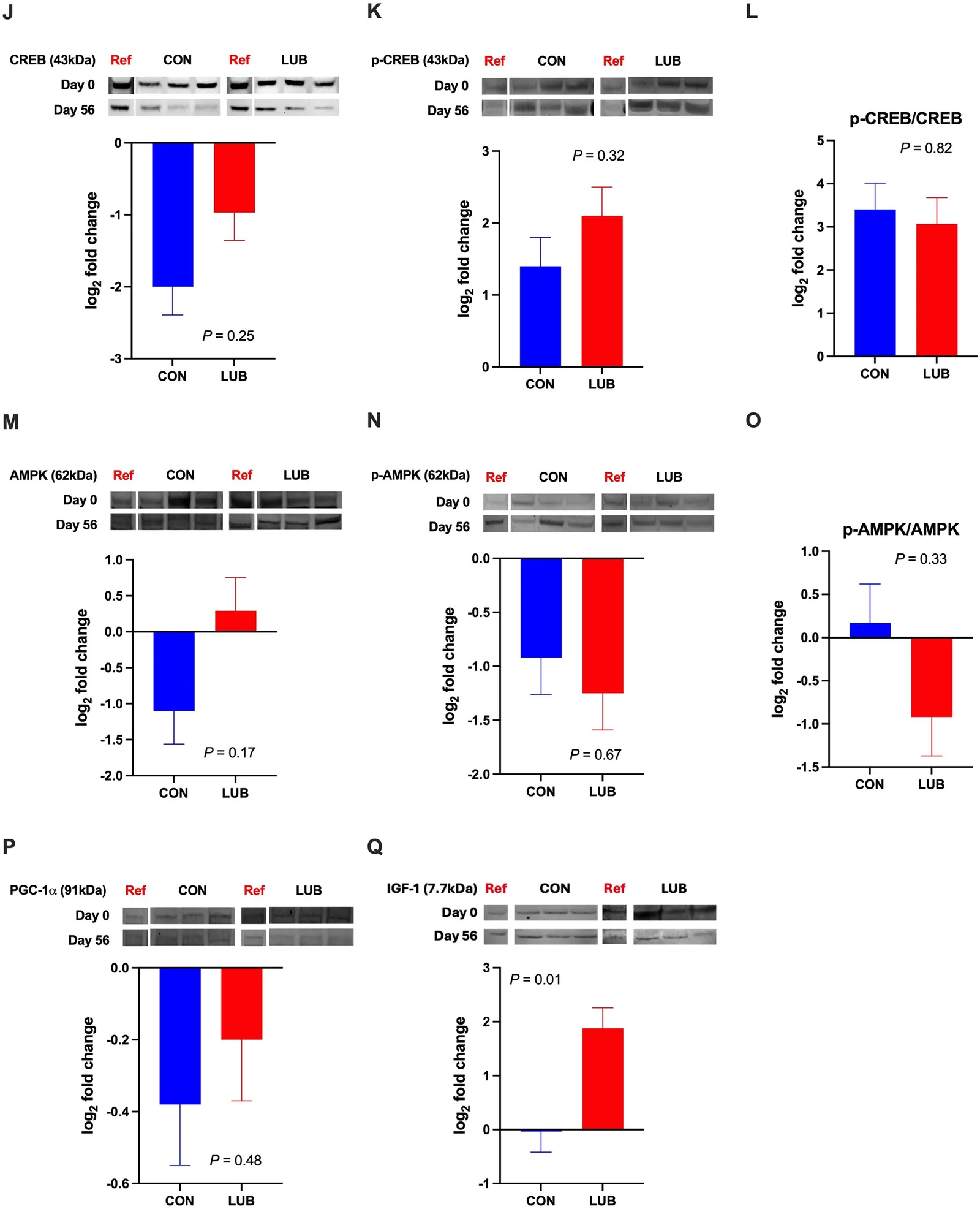
Western blot analyses of anabolic and energy-sensing signaling proteins in longissimus muscle of finishing beef steers supplemented with lubabegron (LUB) or fed a control diet (CON). Representative immunoblots and quantification of log₂-transformed fold changes from d 0 to 56 are shown for CON (blue) and LUB (red) groups. Protein abundance was normalized to total protein stain, and an internal reference sample (Ref) was included on each gel to correct for inter-gel variation. Bar graphs represent least squares means ± SEM. *P*-values reflect treatment effects on changes in protein abundance from d 0 to 56, analyzed using a linear mixed-effects model with pen as a random effect and Šidák adjustment. Akt signaling: total Akt (A; *P* = 0.52), phospho-Akt (p-Akt; B; *P* = 0.15), and p-Akt/Akt ratio (C; *P* = 0.17). mTOR signaling: total mTOR (D; *P* = 0.54), phospho-mTOR (p-mTOR; E; *P* = 0.03), and p-mTOR/mTOR ratio (F; *P* = 0.05). p70S6K signaling: total p70S6K (G; *P* = 0.26), phospho-p70S6K (p-p70S6K; H; *P* = 0.80), and p-p70S6K/p70S6K ratio (I; *P* = 0.21). CREB pathway: total CREB (J; *P* = 0.25), phospho-CREB (p-CREB; K; *P* = 0.32), and p-CREB/CREB ratio (L; *P* = 0.82). AMPK signaling: total AMPK (M; *P* = 0.17), phospho-AMPK (p-AMPK; N; *P* = 0.67), and p-AMPK/AMPK ratio (O; *P* = 0.33). PGC-1α abundance (P; *P* = 0.48) and local IGF-1 protein abundance (Q; *P* = 0.01) are also shown. *n* = 4 pens and 16 steers per treatment.

Total Akt abundance did not differ between CON and LUB at d 56 (*P* = 0.52; Figure 4A). Phosphorylated Akt (p-Akt) and the p-Akt/Akt ratio were numerically greater in LUB but did not differ significantly between treatments (*P* = 0.15 and *P* = 0.17, respectively; Figure 4B and C). No group difference was observed for total mTOR (*P* = 0.54; Figure 4D), whereas p-mTOR was greater in the LUB group (*P* = 0.03; Figure 4E), as was the p-mTOR/mTOR ratio (*P* = 0.05; Figure 4F). Total p70S6K, p-p70S6K, and the p-p70S6K/p70S6K ratio did not differ between treatments (*P *= 0.26, 0.80, and 0.21, respectively; Figure 4G–I).

Total CREB, p-CREB, and the p-CREB/CREB ratio did not differ between treatments (*P* = 0.25, 0.32, and 0.82, respectively; Figure 4J–L). Total AMPK, p-AMPK, and the p-AMPK/AMPK ratio were likewise unaffected by treatment (*P* = 0.17, 0.67, and 0.33, respectively; Figure 4M–O), although total AMPK was numerically greater and the p-AMPK/AMPK ratio numerically lower in LUB. PGC-1α protein abundance did not differ between CON and LUB at d 56 (*P* = 0.48; Figure 4P).

### Circulating and local IGF-1

Serum IGF-1 concentrations did not differ between treatments overall (*P* = 0.66); however, a significant treatment × time interaction was detected (*P* < 0.05; Table 12). At d 28, serum IGF-1 concentrations were similar between CON (444.47 ng/mL) and LUB (451.71 ng/mL). By d 56, serum IGF-1 declined in both groups, with LUB steers exhibiting lower concentrations (358.09 ng/mL) compared with CON (397.97 ng/mL), though pairwise comparisons within each time point did not reach statistical significance. The interaction therefore reflected a divergent temporal pattern rather than a sustained treatment effect on circulating IGF-1.

In contrast, IGF-1 protein abundance in LM tissue (local IGF-1) was greater in LUB-supplemented steers compared with CON at d 56 (*P* = 0.01; Figure 4Q).

### mtDNA abundance of LM

**Figure 5:**
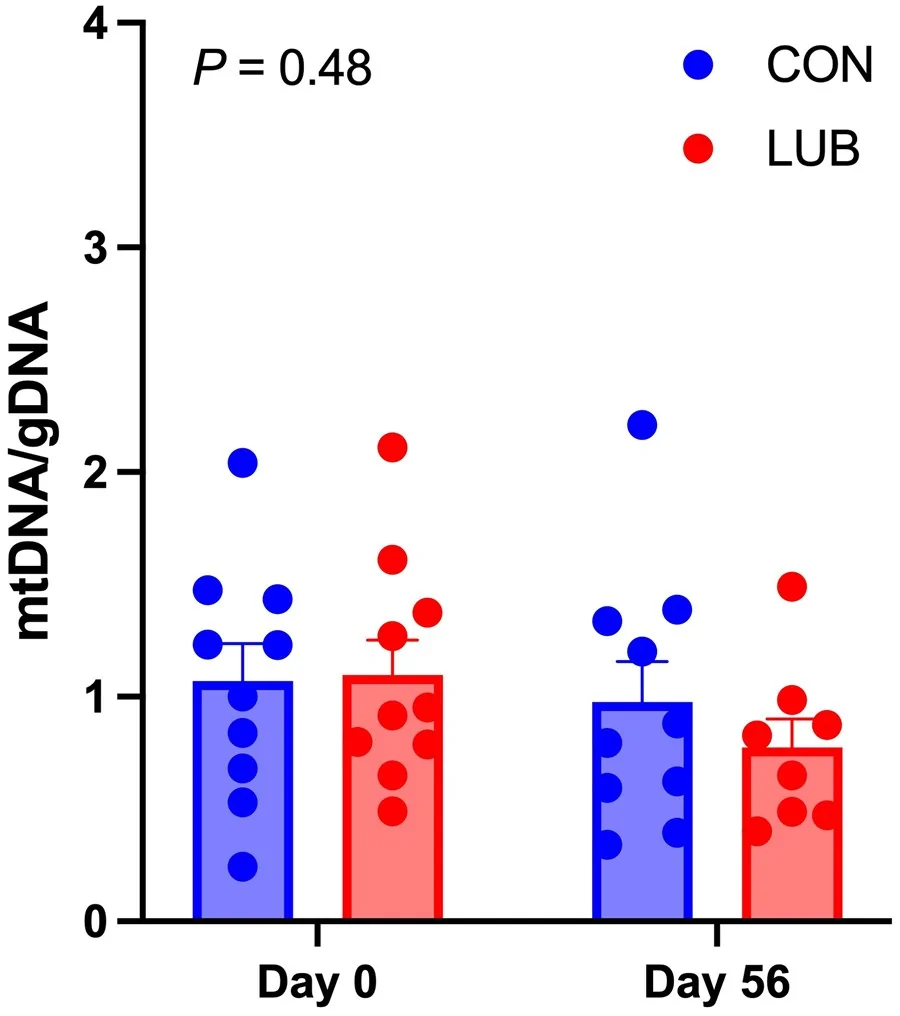
Mitochondrial DNA abundance (mtDNA:gDNA) in skeletal muscle of steers fed control (CON) or lubabegron (LUB) for 56 d. Bars represent means ± SEM, and individual steer observations are shown as points. Day 0 values are presented descriptively, and the *P*-value represents the baseline-adjusted treatment effect at d 56 (*P* = 0.48).

mtDNA abundance, expressed as the mtDNA: gDNA ratio, did not differ between CON and LUB steers at d 56 after adjustment for the corresponding d 0 value (*P* = 0.48; Figure 5).

## Discussion

The findings should be interpreted in light of the limited pen-level replication (*n* = 4 pens per treatment), variation in delivered LUB intake, and the common previously implanted background. Accordingly, the mechanistic findings should be considered exploratory and associative rather than causal.

### Growth performance

LUB supplementation increased ADG and G:F, consistent with previous reports. Across prior studies in which LUB was supplemented at doses ranging from 1.38 to 5.5 mg/kg DM, ADG improvements of 2.6–12.6% have been reported (Kube et al. 2021; Teeter et al. 2021; Kayser et al. 2024), and G:F improvements have been observed consistently (Kube et al. 2021; Teeter et al. 2021; Edmonds et al. 2024; Kayser et al. 2024). The ADG response observed in the present study (28% relative to CON) exceeded the upper end of the range reported in larger-scale LUB feedlot trials (2.6–12.6%; Kube et al. 2021; Teeter et al. 2021; Kayser et al. 2024). Several factors likely contributed to this magnitude, including the relatively small sample size (*n* = 4 pens per treatment), the controlled research-feedlot environment with low stocking density and uniform initial BW. The TE-200 implant was administered at arrival processing, 58 days before the initiation of dietary treatment, such that the treatment period spanned 57–120 days post-implant. Serum trenbolone and estradiol concentrations following non-coated trenbolone acetate–estradiol implants peak within the first several weeks and decline progressively thereafter, with effective payout for this implant type generally considered complete by approximately 100 days (Smith et al. 2019). The day 56 biopsies, from which the local IGF-1, Akt, mTOR, and CREB data were derived, were therefore collected at 113 days post-implant, when implant-derived steroid exposure had largely dissipated. Because the implant was administered identically to CON and LUB steers, this declining exposure was common to both groups. The divergence in local IGF-1 abundance and Akt/mTOR activation observed at day 56 is therefore not attributable to implant-derived steroid signaling. Residual implant effects on muscle cellularity, including satellite cell number and receptor abundance, cannot be excluded, and the LUB responses reported here should be interpreted as occurring on a previously implanted background.

Because all steers were implanted, the present design does not permit estimation of a LUB × implant interaction, and the LUB effects reported here are conditional on a previously implanted background. This constrains generalization to non-implanted cattle but does not bias the CON versus LUB contrast, as implant status did not covary with dietary treatment. A full 2 × 2 factorial evaluation of LUB and implant status is warranted.

Because all steers were implanted, the responses reported here are superimposed on the established anabolic effects of the trenbolone acetate–estradiol combination. Combined trenbolone acetate and estradiol implants stimulate skeletal muscle growth by increasing muscle protein synthesis, reducing protein degradation, and promoting satellite cell proliferation, effects that are accompanied by increased intramuscular IGF-1 expression (Johnson et al. 1998). Implant-driven improvements in ADG are commonly on the order of 10–20% relative to non-implanted controls, which provides an approximate frame of reference for the magnitude of the additional LUB response. Because the implant was administered identically to CON and LUB steers and its payout had largely dissipated by the d 56 sampling, the CON group represents an implanted baseline, and the CON versus LUB contrast isolates the incremental effect of LUB rather than an implant effect. The possibility that residual implant action modified the muscle substrate on which LUB acted, including baseline IGF-1 tone and satellite cell availability, cannot be excluded, and a factorial evaluation of LUB with and without implant is required to partition these contributions.

Reported effects of LUB on DMI have been less consistent across the literature. A large-scale commercial feedlot trial of approximately 2,880 steers supplemented with 1.5, 3.5, or 5.5 mg/kg DM for 56 days reported increased DMI (Kube et al. 2021), whereas no consistent trend was observed in a 91-day clinical effectiveness trial using 1.38–5.5 mg/kg DM (Teeter et al. 2021). In contrast, DMI was reduced in a dose- and duration-dependent manner in beef steers fed 1.5–5.5 mg/kg DM (Edmonds et al. 2024), and was lower in Holstein steers supplemented with 2.88 mg/kg DM (Kayser et al. 2024). In the present study, LUB at 3.5 mg/kg DM increased DMI by 0.88 kg/day relative to CON. The heterogeneity of DMI responses across the literature suggests that intake effects of LUB are sensitive to cattle type, baseline feeding behavior, and trial conditions rather than reflecting a uniform pharmacological effect on appetite regulation. Expressed relative to mean BW during the trial, DMI averaged approximately 1.8% (LUB) and 1.6% (CON) of BW. These values are modestly below the roughly 2% commonly reported for finishing steers, consistent with the energy-dense, high-moisture corn–based diet and the cold-season feeding conditions at this northern-latitude site; this lower relative intake may have constrained the magnitude of some responses.

Final BW increased by 24.8 kg and HCW by 18.5 kg in LUB-supplemented steers, with no difference in dressing percentage (59.99 vs. 59.42%; *P* = 0.25). Relative to the live-weight response, the HCW response represents a marginal carcass yield of approximately 75%, exceeding the whole-body dressing percentage of about 60%, indicating that the additional weight gain in LUB steers was captured as carcass tissue at least as efficiently as existing body mass. This is consistent with larger-scale LUB and β-adrenergic trials in which HCW responses meet or exceed the proportion expected from the live-weight response (Kube et al. 2021; Teeter et al. 2021; Edmonds et al. 2024; Kayser et al. 2024). The unchanged REA and 12th-rib fat thickness indicate that this additional carcass weight was distributed without a measurable shift in those specific traits at the time of harvest.

### Circulating LUB

LUB was detected at 1.80 nM on d 28 and 1.60 nM on d 56, confirming systemic absorption and sustained exposure throughout the treatment period. The observed concentrations were achieved with a dietary inclusion rate of 3.5 mg/kg DM. While a number of studies have investigated the effects of LUB on growth performance, carcass traits, and environmental outcomes, to our knowledge, no published work has previously reported its quantification in blood.

Boison et al. (2016) reported that following oral administration of ^14^C-labeled zilpaterol hydrochloride, plasma radioactivity increased rapidly, reaching peak concentrations at 12 h in males and 10 h in females, as measured in four Charolais × Salers with an average BW of 295 kg. The corresponding maximum plasma concentrations were 16.8 ng/mL and 22.4 ng/mL of zilpaterol equivalents, respectively.

In a study by Tang et al. (2014), ractopamine was administered intraruminally to three culled Holstein dry cows (average BW ∼625 kg) at a dose of 1,025 mg/hd/d for 5 consecutive days. Peak plasma concentrations were 6.10 ng/mL (unhydrolyzed) and 6.27 ng/mL (after β-glucuronidase hydrolysis) on the final day of treatment, with levels falling below the quantification limit (0.2 ng/mL) by withdrawal day five, indicating rapid systemic clearance.

In our study, LUB reached serum concentrations of 1.80 nM on d 28 and 1.60 nM on d 56, corresponding to approximately 0.49–0.55 ng/mL. These values are markedly lower than peak plasma levels of zilpaterol or ractopamine reported in earlier pharmacokinetic studies, yet still sufficient to trigger measurable physiological and molecular changes. The measurable physiological and molecular responses observed despite low circulating concentrations suggest that LUB is biologically active at nanomolar levels *in vivo*. These comparisons should be interpreted cautiously, however, given differences in route of administration, animal type, and dosing regimen across studies; the pharmacokinetic values reported for zilpaterol and ractopamine were derived from controlled bolus or intraruminal administration protocols, whereas LUB concentrations in the present study reflect steady-state serum levels under continuous dietary supplementation.

Serum concentrations may be slightly higher due to analyte release during clotting (Yu et al. 2011); therefore, the observed differences in circulating levels likely underestimate the true disparity in systemic exposure between LUB and the other compounds, rather than being attributable to sample type.

### Serum parameters

Supplementation with LUB influenced several blood parameters indicative of protein metabolism and metabolic homeostasis. Increases in serum creatinine and total protein in LUB-fed steers are consistent with the known effects of β-AA, which promote lean tissue accretion. Similar responses have been reported with zilpaterol, which elevated serum creatinine and reduced plasma urea nitrogen, reflecting increased muscle protein deposition and altered nitrogen utilization (Hales et al. 2016; Antonelo et al. 2020). In the current study, while creatinine was elevated, urea nitrogen did not consistently decline, possibly due to differences in compound-specific mechanisms or dietary nitrogen levels. Ractopamine has also been shown to increase nitrogen retention and, under certain dietary conditions, raise total serum protein (Eisemann et al. 2022), further supporting the protein-accumulating effects observed with LUB. The absence of a decline in serum urea nitrogen may also reflect the moderate DMI and the crude protein concentration of the finishing diet; at this intake and dietary nitrogen supply, greater amino acid incorporation into muscle protein need not produce a detectable reduction in circulating urea nitrogen.

The tendency for greater TCO₂ together with the lower anion gap in LUB steers may suggest a mild shift in acid–base status. However, the physiological significance of these changes remains uncertain because the values were within the expected range for healthy cattle (Kaneko et al. 2008). The increase in serum cholesterol may reflect altered lipid metabolism. Conventional β-AA alter nutrient partitioning and reduce lipid deposition in cattle (Johnson et al. 2014), but the mechanism underlying the cholesterol response to LUB remains unclear. The concurrent increase in serum cholesterol may reflect systemic alterations in lipid handling and is consistent with the transcriptional repression of genes involved in lipid transport and storage in muscle.

The lack of corresponding changes in AST, GGT, or CK, together with the transient nature of the ALP response, provided no clear evidence of liver or muscle damage. Although LUB altered several blood-based metabolic markers, the measured values remained within the ranges expected for healthy cattle (Kaneko et al. 2008). The small increases in total and indirect bilirubin may reflect altered hepatic bilirubin clearance or increased heme breakdown (McSherry et al. 1984; Kaneko et al. 2008). However, because a complete blood count and specific markers of hemolysis were not measured, the cause of the bilirubin response could not be determined.

### Carcass traits

In this study, LUB supplementation significantly increased HCW, while other carcass traits such as dressed yield, REA, 12th-rib fat thickness, KPH fat, and calculated yield grade were not affected. These findings are partially consistent with previous studies. For instance, Teeter et al. (2021) and Kayser et al. (2024) reported an increase in HCW following LUB supplementation. However, unlike our results, those studies additionally observed improvements in dressing percentage and REA, along with reductions in 12th-rib fat thickness.

Marbling score was not influenced by LUB in the current study. In contrast, several earlier reports have documented reductions in marbling with LUB feeding (Kube et al. 2021; Teeter et al. 2021; Edmonds et al. 2024; Kayser et al. 2024). These potential mechanisms are addressed in the context of transcriptomic data below. The absence of a marbling effect in the present study may reflect the limited replication, as well as differences in supplementation duration, genetic background, or baseline fatness relative to trials reporting reduced marbling. At the transcriptomic level, adipogenic and lipid-metabolism pathways were downregulated in LUB muscle, a pattern consistent with reduced adipogenic programming that was not accompanied by a measurable change in marbling or intramuscular fat at this stage.

### Proximate composition and texture analysis

Corona et al. (2025) evaluated the effects of LUB supplementation on meat quality and found that steers fed 3.5–5.5 mg/kg for 28 to 84 d had higher WBSF values, along with reduced tenderness and juiciness. Although LUB-fed steaks at 14 days postmortem (3.74 kg) remained within the USDA Certified Very Tender classification (≤3.9 kg; ASTM F2925-11), interpretation of this categorical reference warrants caution. The ASTM thresholds were derived from regression analyses anchored in consumer panel data collected in the late 1980s and early 1990s ( Smith et al. 1982; Shackelford et al. 1991), and average industry WBSF values have declined substantially over the subsequent three decades (Brooks et al. 2000; Guelker et al. 2013; Martinez et al. 2017). In the most recent reevaluation of consumer tenderness perception, Martinez et al. (2023) reported that the average WBSF of US top loin steaks had decreased to 2.57 kg and proposed updated thresholds of approximately 2.20 kg for “very tender or greater” and 2.85 kg for “moderately tender” under modern consumer baselines. By these contemporary benchmarks, CON steaks in the present study (2.76 kg at 14 d) would fall within the “moderately tender” range, whereas LUB steaks (3.74 kg) would shift into the “slightly tender” range, indicating a perceptible category-level difference rather than equivalence within a single legacy classification. Furthermore, the magnitude of the WBSF difference between treatments at 14 days (0.98 kg) approaches the threshold of consumer detectability reported in trained and untrained sensory panels (∼0.5–1.0 kg; Miller et al. 2001; Platter et al. 2003), and the relative increase of approximately 35% is consistent with the compositional shift toward greater protein and reduced intramuscular fat observed in LUB-fed steers. Although objective shear force was greater in LUB steers, whether this difference is large enough to affect consumer tenderness perception is not established by the present data and warrants direct sensory evaluation.

### RNA sequencing and transcriptional remodeling

At d 56, a total of 643 DEGs were identified, with 523 upregulated and 120 downregulated in the LUB group compared to controls. These transcriptional shifts suggest that LUB supplementation alters muscle phenotype by modulating gene networks involved in growth, metabolism, and cellular stress adaptation.

Upregulated genes included several well-established regulators of myogenesis and muscle fiber specification, including *MYF6, MYH1, SRF, KLF5*, and *SOX6* (Asfour et al. 2018). *MYF6*, also known as *MRF4*, is a terminal myogenic transcription factor that acts downstream of *SRF* and *KLF5*, which enhance *MYOD1*- and *MYOG*-mediated transcriptional activation (Shirakawa et al. 2022). SRF has been shown to regulate hypertrophic signaling through paracrine mechanisms involving satellite cells (Guerci et al. 2012), while *SOX6* promotes fast-twitch fiber gene expression by repressing slow-type fiber transcriptional programs (An et al. 2011). *PLPP7*, which is enriched in fast-twitch muscle fibers and transcriptionally regulated by *MYOD1* and *MYOG*, was also upregulated; this protein has demonstrated roles in maintaining myonuclear integrity and chromatin organization in skeletal muscle (Ramirez-Martinez et al. 2021). These transcriptional changes are consistent with activation of a myogenic program favoring satellite cell differentiation and myofiber remodeling in response to LUB supplementation.

LUB supplementation also altered the metabolic gene expression profile of skeletal muscle. Genes encoding key glycolytic enzymes, including *PFKM* and *PKM*, were upregulated in LUB-treated steers. *PFKM* encodes phosphofructokinase, the rate-limiting enzyme of glycolysis, and its upregulation alongside *PKM* indicates greater glycolytic capacity and anaerobic ATP production potential (García et al. 2009). At the same time, *PGC-1α* mRNA was transiently suppressed in LUB-treated steers at d 28 relative to d 0, while CON steers showed an increase over the same interval (treatment × time interaction), suggesting an early attenuation of oxidative metabolic programming in LUB-supplemented muscle. This difference was not sustained at d 56, indicating that the effect was transient rather than a permanent reprogramming of oxidative capacity. While the concurrent upregulation of *MYH1* and *SOX6* is directionally consistent with fast-twitch fiber characteristics, fiber-type composition was not directly assessed by histology in the present study, and this interpretation remains provisional. Nonetheless, a more glycolytic intramuscular metabolic environment is generally associated with greater lean accretion potential but reduced tenderness and lower intramuscular fat content (Wang et al. 2024), which is consistent with the greater WBSF values and tendency toward lower crude fat content observed in LUB-fed steers. The reductions in marbling score reported across multiple LUB trials (Kube et al. 2021; Teeter et al. 2021; Edmonds et al. 2024; Kayser et al. 2024) may partly reflect this pattern.

*MAP2K2* and *MAPK6* were also upregulated in LUB-treated steers, indicating activation of the mitogen-activated protein kinase (MAPK)/extracellular signal-regulated kinase (ERK) signaling cascade. *MAP2K2* encodes *MEK2*, which phosphorylates and activates ERK1/2 to facilitate *MYOD1* and muscle growth, while MAPK6 (ERK3) has been implicated in cytoskeletal organization and transcriptional regulation during muscle development (Soulez et al. 2022). The upregulation of these genes coincided with a tendency for greater *IGF-1* mRNA expression by RT-qPCR, which is consistent with evidence that IGF-1 activates both the PI3K/Akt and MAPK/ERK pathways in parallel to promote skeletal muscle hypertrophy (Fuentes et al. 2011; Khan et al. 2025). The relevance of this observation to the proposed IGF-1-mediated anabolic mechanism is addressed in the following section.

MSTN, a member of the TGF-β superfamily, is a well-established negative regulator of skeletal muscle mass that suppresses satellite cell proliferation and inhibits protein synthesis through Smad2/3-mediated repression of myogenic regulatory factors (McPherron et al. 1997; Lee 2004). In the present study, *MSTN* mRNA expression increased over time regardless of treatment, with no treatment or treatment × time interaction effects detected by RT-qPCR. This temporal increase may represent a homeostatic feedback response to progressive muscle accretion during the 63-d finishing period. Consistent with this interpretation, Winbanks et al. (2012) demonstrated that mTORC1 activation induces MSTN expression in skeletal muscle as a negative feedback mechanism to constrain excessive hypertrophy, suggesting that the progressive activation of mTOR signaling observed in LUB-supplemented steers may have contributed, in part, to the time-dependent rise in MSTN transcript abundance. Similarly, IGF-1-driven activation of the PI3K/Akt/mTOR axis has been shown to co-occur with increased MSTN expression during periods of active muscle remodeling, where MSTN functions as an autocrine brake limiting the magnitude of hypertrophic response (Sandri 2008; Schiaffino et al. 2013). The absence of a treatment effect on MSTN mRNA, despite robust activation of anabolic signaling in LUB steers, suggests that this negative feedback was engaged proportionately across groups as a function of the growth phase rather than as a compound-specific response.

In contrast, several key regulators of lipid metabolism were suppressed by LUB supplementation. Lipoprotein lipase (*LPL*), which hydrolyzes circulating triglycerides to liberate fatty acids for uptake, was downregulated. Likewise, *PLIN5*, a lipid-droplet-associated protein that controls interactions with mitochondria and lipases in oxidative fibers (Bindesb et al. 2013), and *FABP3*, which mediates intracellular fatty acid trafficking toward mitochondria (Smathers and Petersen 2011), were downregulated. Overall, the downregulation of these genes indicates a diminished capacity for fatty acid uptake, storage, and mobilization within skeletal muscle (Furuhashi and Hotamisligil 2008). The transient suppression of *PGC-1α* in LUB-treated steers at d 28, combined with sustained downregulation of *PPARα*, further supports a shift away from oxidative lipid metabolism toward glycolytic pathways. Additional regulatory genes were also significantly altered by LUB supplementation. *ZBTB16* (also known as *PLZF*) is a zinc finger transcription factor that has been shown to inhibit adipogenic differentiation by repressing PPARγ activity and maintaining cells in an undifferentiated state (Wei et al. 2013). Transmembrane protein (*TMEM64*) has been implicated in modulating intracellular calcium signaling and mTOR activity, which are key pathways in energy sensing and adipocyte metabolism (Cai et al. 2016). Nuclear receptor coactivator 1 (*NCOA1*) enhances transcriptional activity of several nuclear receptors, including PPARs and glucocorticoid receptors; Increased *NCOA1* expression may reflect compensatory adaptation to disrupted lipid signaling (Chen et al. 2010). Circulating non-esterified fatty acids (NEFA) were not measured in the present study. Given the coordinated downregulation of *LPL*, *FABP3*, and *PLIN5*, which together govern fatty acid uptake, trafficking, and storage in muscle, LUB may alter systemic NEFA availability and partitioning; consistent with this, Henderson et al. (2026) reported reduced serum NEFA in LUB-supplemented steers. Direct measurement of serum NEFA is warranted to reconcile these transcriptional changes with reported effects of β-adrenergic ligands on lipid mobilization.

These observations are in line with the mechanistic findings of Hwang et al. (2021, 2022), who used an *in vitro* approach showing that LUB acts as a β_1_/β_2_-AR antagonist in adipose tissue, blocking downstream signaling (cAMP, PKA) and lipolytic gene activation. Studies reported that adipocytes exhibit lower β-AR expression and weaker lipolytic responses, which may explain the site-specific effects of LUB on fat metabolism and reduced marbling *in vivo*. The transcriptional data indicated that LUB supplementation is associated with suppressed adipogenic signaling and enhanced myogenic and glycolytic programs. The changes in expression of *ZBTB16, TMEM64*, and *NCOA1* also indicate a coordinated response that may help reduce lipid accumulation in muscle.

Ras-related GTP-binding D (*RRAGD*), a critical upstream regulator of the mTORC1 pathway, was upregulated, suggesting that LUB enhances amino acid-dependent nutrient-sensing (Gollwitzer et al. 2022). Activation of RRAGD facilitates the recruitment of mTORC1 to the lysosomal membrane in response to intracellular amino acid availability, thereby promoting protein synthesis and cellular growth (Gatica and Klionsky 2017).

Simultaneously, several genes involved in proteostasis and stress adaptation were also upregulated. *HSPB2* and *HSP90AA1* are molecular chaperones that stabilize unfolded proteins and protect muscle cells from proteotoxic stress, particularly during periods of high protein turnover or mechanical load (Pomella et al. 2023). *ATF4*, a transcription factor activated during endoplasmic reticulum and integrated stress responses, regulates amino acid metabolism and antioxidant defense pathways (Harding et al. 2003; Ryoo 2024). EEF2K, a kinase that controls translation elongation via phosphorylation of eEF2, acts as a metabolic checkpoint to modulate protein synthesis under energy-limited conditions (Leprivier et al. 2013).

### Potential mechanisms of Akt/mTOR activation in skeletal muscle following LUB supplementation

**Figure 6:**
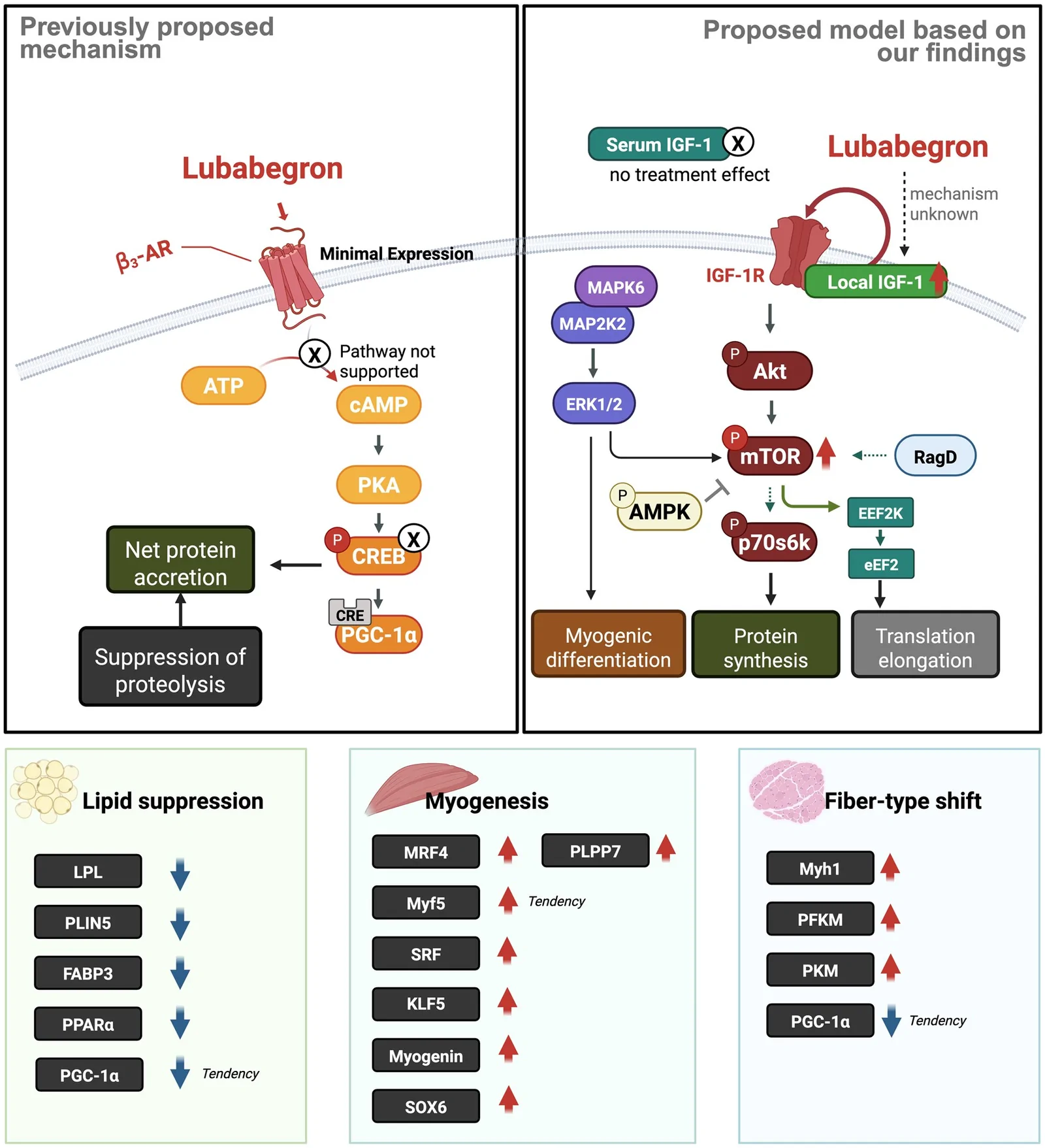
Previously proposed and revised mechanistic model of lubabegron action in bovine skeletal muscle. (Left panel) Previously proposed mechanism. Lubabegron was hypothesized to exert anabolic effects in skeletal muscle through β_3_-adrenergic receptor (β_3_-AR) activation, initiating the cAMP–PKA–CREB signaling cascade to promote net protein accretion via suppression of proteolysis and transcriptional activation of downstream targets. However, β_3_-AR transcript abundance in longissimus muscle (LM) was minimal and undetectable in 40.6% of samples, and neither phospho-CREB abundance nor the p-CREB/CREB ratio differed between treatment groups, providing no support for this pathway as the primary driver in LM. Pathway marked with ✕ indicates the classical β_3_-AR/cAMP/PKA/CREB axis for which the present data provide no direct support as a primary driver in LM. A contribution of β_3_-AR to local IGF-1 upregulation through alternative signaling routes has not been experimentally excluded. (Right panel) Revised model based on present findings. Lubabegron supplementation significantly elevated local IGF-1 protein abundance within LM without a corresponding increase in circulating serum IGF-1 concentrations, suggesting autocrine and paracrine IGF-1 production within the muscle microenvironment. Locally produced IGF-1 is proposed to activate the IGF-1 receptor (IGF-1R), engaging the PI3K/Akt/mTOR signaling axis. Increased phosphorylation of mTOR was confirmed by Western blot, with numerically greater Akt phosphorylation. Downstream, mTOR promotes protein synthesis through p70S6K activation and regulates translation elongation via EEF2K-mediated phosphorylation of eEF2. Total AMPK abundance was numerically greater in lubabegron-supplemented steers and relative AMPK activity (p-AMPK/AMPK) numerically lower, although these differences were not statistically significant. Upregulation of MAP2K2 and ERK1/2, components of the parallel MAPK/ERK pathway downstream of IGF-1R, further supports engagement of the IGF-1 signaling network. RagD (RRAGD), an upstream regulator of mTORC1 recruitment in response to amino acid availability, was among the most significantly upregulated genes in RNA sequencing data. The dashed arrow from lubabegron to local IGF-1 indicates a proposed indirect mechanism, the nature of which was not directly determined in the present study. (Bottom panels) Transcriptional outcomes associated with lubabegron supplementation as identified by RNA sequencing and RT-qPCR. Lipid suppression: downregulation of genes governing fatty acid uptake (LPL, FABP3), lipid droplet dynamics (PLIN5), and oxidative metabolism (PPARα, PGC-1α). Myogenesis: upregulation of myogenic regulatory factors (MRF4, MYF5, MYOG), structural regulators (SRF, KLF5, SOX6), and nuclear envelope protein PLPP7, collectively supporting enhanced satellite cell differentiation and myofiber remodeling. Fiber-type shift: upregulation of fast-twitch myosin heavy chain isoform MYH1 and glycolytic enzymes (PFKM, PKM), with a tendency for reduced MYH7 (slow-twitch isoform) expression and transient suppression of PGC-1α mRNA, consistent with a shift toward a glycolytic muscle phenotype. Upward arrows indicate upregulation or increased phosphorylation relative to CON. Downward arrows indicate downregulation or decreased abundance. Solid arrows indicate pathways supported by experimental data from the present study. Dashed arrows represent proposed or indirect connections. Inhibitory symbols denote attenuated signaling. This figure was created with BioRender (BioRender.com).

To investigate the cellular mechanisms underlying the skeletal muscle response to LUB supplementation, we initially hypothesized that LUB activates β_3_-AR in skeletal muscle, triggering cAMP–PKA–CREB signaling and downstream anabolic pathways that favor protein accretion (Figure 6). This hypothesis was motivated by LUB’s established pharmacological profile as a selective β_3_-AR agonist with concurrent antagonism at β_1_- and β_2_-AR subtypes (Hwang et al. 2021), and by evidence that all three β-AR subtypes can couple to Gs proteins and activate the cAMP–PKA cascade (Anderson et al. 2005). However, β_3_-AR transcript levels in LM biopsies were minimal and undetectable in 13 of 32 samples. It should be noted, nevertheless, that transcript abundance reflects synthesis rather than protein abundance; given β_3_-AR’s known resistance to agonist-induced internalization and desensitization (Nantel et al. 1993), preexisting receptor protein may remain functional at the membrane independent of ongoing transcription. Accordingly, low ADRB3 mRNA does not preclude functional receptor presence in this tissue. Furthermore, although Dilger et al. (2021) proposed that β_3_-AR activation may promote skeletal muscle hypertrophy, a direct mechanistic link between β_3_-AR and mTOR activation in muscle has not been experimentally established, and our data do not provide direct support for this axis as the primary driver in LM.

If β_3_-AR were the primary driver of anabolic signaling in LM, activation of the cAMP–PKA axis would be expected to produce measurable increases in CREB phosphorylation. However, neither p-CREB abundance nor the p-CREB/CREB ratio differed between treatment groups, providing little support for sustained β_3_-AR–mediated cAMP–PKA–CREB signaling in this tissue. Furthermore, if LUB simultaneously antagonizes β_1_- and β_2_-AR, the net adrenergic effect in skeletal muscle, where β_1_- and β_2_-AR are the predominant subtypes, would be suppressive rather than stimulatory. This antagonistic profile alone cannot readily account for the consistent and robust anabolic phenotype, including improved ADG, increased HCW, and elevated muscle protein content, observed in this and multiple prior studies (Kube et al. 2021; Teeter et al. 2021; Edmonds et al. 2024; Kayser et al. 2024). Collectively, these observations indicate that the classical β_3_-AR/cAMP/PKA/CREB axis does not appear to be the primary driver in LM, and that an alternative or complementary upstream mechanism is implicated. Whether β3-AR engagement contributes to local IGF-1 upregulation through non-classical signaling, such as via CREB coactivators Crtc2/3, which can activate CRE-dependent transcription independently of p-CREB phosphorylation (Bruno et al. 2014), remains to be determined and should be addressed using selective β3-AR antagonism in primary bovine myotube models.

The signaling data collectively support an alternative, IGF-1-mediated mechanism. Despite minimal β_3_-AR expression in LM, WB analyses revealed clear activation of the Akt/mTOR pathway in LUB-supplemented steers: p-mTOR abundance and the p-mTOR/mTOR ratio were significantly elevated in the LUB group, whereas total and phosphorylated Akt did not differ significantly, although p-Akt and the p-Akt/Akt ratio were numerically greater in LUB. The activation of this canonical anabolic axis in the absence of evidence for classical β_3_-AR/cAMP/PKA/CREB signaling is consistent with an upstream signal that operates at least partially independently of this adrenergic cascade, though a contribution of β_3_-AR through alternative downstream routes cannot be excluded.

The most compelling candidate is locally produced IGF-1. WB analysis demonstrated significantly greater IGF-1 protein abundance in LM of LUB-supplemented steers at d 56, providing direct evidence for enhanced local IGF-1 production within the muscle microenvironment. This finding was corroborated at the transcript level by a tendency for increased *IGF1* mRNA expression by RT-qPCR and, at the pathway level, by RNA sequencing data revealing coordinated upregulation of established IGF-1 signaling mediators. *RRAGD*, a Rag GTPase involved in amino acid-dependent recruitment of mTORC1 to the lysosomal surface (Sancak et al. 2010; Gollwitzer et al. 2022), was among the most significantly upregulated genes. MAP2K2 and MAPK6, components of the MAPK/ERK pathway activated in parallel with PI3K/Akt downstream of the IGF-1 receptor (Huang et al. 2010), were upregulated. Additional downstream effectors of IGF-1 signaling, including NCOA1, which modulates transcriptional activity of nuclear receptors, and EEF2K, which regulates translational elongation capacity (Wang et al. 2001), were also elevated, further supporting broad engagement of the IGF-1 signaling network.

The source of this elevated IGF-1 appears to be local rather than systemic. IGF-1 exerts anabolic effects through two functionally distinct mechanisms: hepatic-derived IGF-1 mediates systemic endocrine signaling through the circulation, whereas locally produced IGF-1 within skeletal muscle acts in an autocrine and paracrine manner to regulate satellite cell activity, myofiber hypertrophy, and protein synthesis (Adams and McCue 1998; Adams 2002; Schiaffino et al. 2013). Serum IGF-1 concentrations did not differ between treatments overall, though a significant treatment × time interaction was detected, reflecting divergent temporal trajectories rather than a sustained elevation of circulating IGF-1 in LUB-fed steers. The dissociation between significantly elevated local muscle IGF-1 protein and the absence of a consistent treatment effect on serum IGF-1 supports the interpretation that the Akt/mTOR activation observed in this study was driven primarily by autocrine and paracrine IGF-1 signaling within the muscle microenvironment, rather than by increased hepatic output. To our knowledge, this is the first report of elevated local IGF-1 protein in bovine skeletal muscle with LUB despite concurrent β_1_/β_2_-AR antagonism. Because local IGF-1 induction is also observed with classical β-agonists (Awede et al. 2002), this finding identifies IGF-1/Akt/mTOR as a convergent downstream node and offers a coherent explanation for the muscle responses reported with LUB across independent studies. Although the present study does not provide direct causal evidence that locally produced IGF-1 is responsible for Akt/mTOR activation, the co-occurrence of elevated local IGF-1, activated Akt/mTOR signaling, and coordinated upregulation of established IGF-1 downstream mediators (*RRAGD, MAP2K2, MAPK6*) is consistent with this interpretation. Direct confirmation will require targeted IGF-1R blockade or knockdown. β-adrenergic signaling has been reported to stimulate local IGF-1 production in skeletal muscle (Awede et al. 2002; Gonçalves et al. 2019), and CREB-mediated transcriptional activation in myocytes can be induced by β-agonists through the obligate coactivators Crtc2 and Crtc3 independently of PKA-mediated CREB phosphorylation (Bruno et al. 2014). Accordingly, unchanged p-CREB abundance in the present study does not definitively exclude a contribution of residual β_3_-AR signaling to local IGF-1 upregulation, as Crtc-mediated transcriptional activation would not be reflected in p-CREB levels alone.

An additional, non-mutually exclusive possibility is that LUB acts in part by modifying systemic insulin action. Selective β₃-AR agonists were originally developed as insulin-sensitizing agents for type 2 diabetes, and the β₃-AR agonist mirabegron improves insulin sensitivity and glucose homeostasis in humans (Finlin et al. 2020; O’Mara et al. 2020). Consistent with this pharmacology in cattle, Henderson et al. (2026) reported that LUB supplementation increased insulin sensitivity, indexed by the revised quantitative insulin sensitivity check index, and lowered circulating insulin, glucose, and non-esterified fatty acids in finishing steers, in agreement with the reduced insulin area under the curve observed during a glucose tolerance challenge in the LUB approval data (FDA 2018). The insulin receptor (IR) and IGF-1R share extensive structural homology and form functional IR/IGF-1R hybrid receptors in skeletal muscle, all of which signal through IRS-1/2-PI3K-Akt-mTOR. Enhanced insulin action at IGF-1R or IR/IGF-1R hybrids could therefore contribute to the Akt/mTOR activation observed here without an increase in circulating IGF-1. In the present study, serum glucose did not differ between treatments; serum insulin and direct indices of insulin sensitivity were not measured, so this route cannot be evaluated from our data. Distinguishing locally produced IGF-1 from insulin-driven signaling at the IGF-1R will require quantification of circulating insulin and insulin sensitivity together with receptor-selective blockade in bovine myotube models.

Energy-sensing pathways were also examined. Total AMPK, p-AMPK, and the p-AMPK/AMPK ratio did not differ significantly between treatments, although total AMPK was numerically greater and the p-AMPK/AMPK ratio numerically lower in LUB-fed steers. Because AMPK acts as a negative regulator of mTORC1 under energetic deficit (Hardie 2011), these directional patterns are compatible with, but do not establish, an attenuation of AMPK-mediated inhibitory signaling in LUB-treated muscle. Similarly, total p70S6K, a direct substrate of mTORC1, did not differ significantly between treatments. Taken together, the data support a model in which LUB supplementation is associated with increased local IGF-1 abundance in skeletal muscle and with activation of the PI3K/Akt/mTOR axis, whereas any contribution of altered AMPK signaling remains to be established. This proposed mechanism is distinct from that of traditional beta-adrenergic agonists such as ractopamine and zilpaterol, which are thought to promote lean tissue accretion primarily through direct β1- and β_2_-AR activation and subsequent cAMP-PKA-mTOR engagement, and may explain why LUB produces comparable anabolic outcomes through a divergent molecular route.

Several limitations of the present study warrant consideration. Treatment was applied at the pen level, and although individual steers were sampled, the number of independent experimental units was small (*n* = 4 pens per treatment; 16 steers per treatment), which limits statistical power and the generalizability of the mechanistic findings; independent replication in larger trials is needed to confirm the proposed IGF-1-centered signaling model. In addition, analyzed LUB concentrations in the TMR varied across the feeding period and estimated delivered intake (mean, 31.5 mg/steer/day) was below the 36 mg/steer/day target, indicating that the responses reported here were obtained at a somewhat lower effective dose than intended. Moreover, mechanistic investigations of LUB in bovine skeletal muscle remain scarce, and the present study should be viewed as hypothesis-generating with respect to the underlying molecular pathway.

Environmental and genetic context may also have influenced the observed responses. This trial was conducted during winter months at a northern latitude (Michigan, USA), where ambient temperatures fell below the thermoneutral zone for cattle. Cold exposure is known to activate the sympathetic nervous system and increase energy expenditure, and there is evidence that the thermal environment can modulate the GH–IGF-1 axis and alter nutrient partitioning patterns in ruminants (Sarko et al. 1994). It is therefore possible that the heightened IGF-1 response observed in this study was, in part, facilitated by cold-induced physiological adaptations. In contrast, the majority of published LUB studies were conducted at feedlots in the southern United States, where warmer climates prevail, and cattle populations may include a greater proportion of Bos indicus-influenced genetics, which differ from Angus-influenced steers in AR density and responsiveness (Johnson et al. 2014). Whether the candidate mechanism identified here, local IGF-1 upregulation and Akt/mTOR activation, is consistent across thermal environments, seasons, and breed compositions should be examined in future studies.

## Conclusion

Supplementation with LUB at 3.5 mg/kg DM improved ADG, DMI, and feed efficiency in finishing beef steers, accompanied by changes in muscle composition. These outcomes were accompanied by increased HCW and greater crude protein content in LM, with reduced tenderness, reflecting coordinated changes in muscle tissue accretion and composition.

At the molecular level, transcriptomic profiling and targeted gene expression analyses revealed extensive remodeling of skeletal muscle, characterized by upregulation of myogenic regulatory factors, glycolytic enzymes, and genes associated with fast-twitch fiber specification, with suppression of lipid metabolism and adipogenic gene networks. These transcriptional patterns support a nutrient repartitioning effect that favors lean tissue deposition over intramuscular lipid accumulation. Protein-level analyses supported enhanced mTOR signaling, with significant increases in p-mTOR and the p-mTOR/mTOR ratio, consistent with a shift toward an anabolic cellular state.

β_3_-AR transcript was undetectable in 13 of 32 LM samples (40.6%), and CREB phosphorylation was unchanged by LUB, providing no support for the classical β_3_-AR/cAMP/PKA/CREB axis as the primary driver in LM. Low transcript abundance, however, does not preclude the presence of functional β_3_-AR protein, and whether β_3_-AR contributes to the observed IGF-1 elevation through alternative signaling routes warrants direct investigation.

To our knowledge, this is the first report of greater local IGF-1 protein abundance in bovine skeletal muscle following LUB supplementation without a corresponding sustained increase in circulating IGF-1. This dissociation between local and systemic IGF-1 is consistent with a proposed model in which local muscle IGF-1 may contribute to enhanced mTOR signaling. Coordinated upregulation of downstream mediators, including RRAGD, MAP2K2, and MAPK6, provides additional associative support for involvement of the IGF-1 signaling network. However, causality between greater local IGF-1 abundance and mTOR activation was not established in the present study, and the involvement of Akt, β₃-AR, or other upstream receptors cannot be determined from these data. Confirmation of this proposed mechanism will require receptor-selective blockade or knockdown experiments.

## Supplementary Material

skag281_Supplementary_Data

## Abbreviations

β-AA: β-adrenergic agonist
β-AR: β-adrenergic receptor
ACTB: actin beta; ADF, acid detergent fiber
ADG: average daily gain
ADRB1–3: β-adrenergic receptors 1–3
ALP: alkaline phosphatase
AMPK: AMP-activated protein kinase
AR: adrenergic receptor
AST: aspartate aminotransferase
BCTRC: Beef Cattle Teaching and Research Center
BW: body weight
cAMP: cyclic adenosine monophosphate
C/EBPα: CCAAT/enhancer-binding protein alpha
CK: creatine kinase
COL1A1: collagen type I alpha 1 chain
CON: Control (non-treated group)
COX2: cytochrome c oxidase subunit II
CREB: cAMP response element-binding protein
Crtc2/3: CREB-regulated transcriptional coactivators 2/3
DDGS: dried distillers grains with solubles
DEG: differentially expressed gene
DM: dry matter
DMI: dry matter intake
ERK: extracellular signal-regulated kinase
FABP3/FABP4: fatty acid binding protein 3/4
FDR: false discovery rate
G:F: gain-to-feed ratio
GGT: gamma-glutamyl transferase
GH: growth hormone
HCW: hot carcass weight
IACUC: Institutional Animal Care and Use Committee
IGF-1: insulin-like growth factor 1
KPH: kidney, pelvic, and heart fat
L*, a*, b*: CIE Lab* color space values (lightness, redness, yellowness)
LM: longissimus muscle
LPL: lipoprotein lipase
LUB: lubabegron
MAPK: mitogen-activated protein kinase
Mcal: megacalorie
MSTN: myostatin
mTOR: mechanistic target of rapamycin
mRNA: messenger RNA
mtDNA: mitochondrial DNA
MSU VDL: Michigan State University Veterinary Diagnostic Laboratory
Na:K: sodium-to-potassium ratio
NCOA1: nuclear receptor coactivator 1
NDF: neutral detergent fiber
NEm: net energy for maintenance
NEg: net energy for gain
p70S6K: p70 ribosomal S6 kinase
PGC-1α: peroxisome proliferator-activated receptor gamma coactivator 1-alpha
PI3K: phosphoinositide 3-kinase
PKA: protein kinase A
PPARα/γ: peroxisome proliferator-activated receptor alpha/gamma
qPCR: quantitative polymerase chain reaction
REA: ribeye area
RIN: RNA integrity number
RNA-seq: RNA sequencing
RRAGD: Ras-related GTP binding D
SDS-PAGE: Sodium dodecyl sulfate polyacrylamide gel electrophoresis
SRF: Serum response factor
TAB1: TGF-β activated kinase 1 binding protein 1
TCO₂: Total carbon dioxide
TDN: total digestible nutrients
TMEM64: transmembrane protein 64
TMR: total mixed ration
T × T: treatment × time interaction
UPLC–MS/MS: ultra-performance liquid chromatography–tandem mass spectrometry
WB: Western blot
WBSF: Warner–Bratzler shear force
ZBTB16: zinc finger and BTB domain containing 16
ZNF423/750: zinc finger protein 423/750
