# Supplementary material for: Local insulin-like growth factor-1 and Akt/mTOR signaling in skeletal muscle of lubabegron-supplemented finishing steers
Source: J Anim Sci. 2026 Sep 2;104:skag281. doi: 10.1093/jas/skag281 (PMC13588578; doi:10.1093/jas/skag281)
Supplement: Supplementary file 1 [file skag281_supplementary_data.docx]

**Table S1.** MS/MS parameters

| Compound | Precursor ion (m/z) | Product ion (m/z) | Cone voltage (V) | Collision energy(eV) |
| --- | --- | --- | --- | --- |
| Lubabegron (quantitative) | 500.40 | 250.08 | 46 | 16 |
| Lubabegron (qualitative) | 500.40 | 209.00 | 46 | 34 |
| Lubabegron (qualitative) | 500.00 | 187.00 | 46 | 34 |
| Telmisartan | 515.20 | 276.10 | 66 | 50 |

**Table S2.** Primary and secondary antibodies used for Western blot analysis of signaling proteins in longissimus muscle of finishing beef steers.

| Antibody target | Primary  /Secondary | Source | Dilution | Manufacturer | Identifier |
| --- | --- | --- | --- | --- | --- |
| mTOR | Primary | Rabbit | 1:1000 | Cell signaling | 2983 |
| p-mTOR (Ser2448) | Primary | Rabbit | 1:1000 | Cell signaling | 2971 |
| Akt | Primary | Mouse | 1:100 | DSHB | CPTC-AKT1-1 |
| p-Akt (Ser473) | Primary | Rabbit | 1:1000 | Cell signaling | 9271 |
| p70^s6k^ | Primary | Rabbit | 1:1000 | Cell signaling | 9202 |
| p-p70^s6k^ (Thr389) | Primary | Rabbit | 1:1000 | Cell signaling | 9234 |
| CREB | Primary | Rabbit | 1:1000 | Cell signaling | 9197 |
| p-CREB (Ser133) | Primary | Rabbit | 1:1000 | Cell signaling | 9198 |
| AMPK | Primary | Rabbit | 1:1000 | Cell signaling | 9532 |
| p-AMPK (Thr172) | Primary | Rabbit | 1:1000 | Cell signaling | 2535 |
| PGC-1α | Primary | Rabbit | 1:1000 | Abcam | ab191838 |
| IGF-1 | Primary | Goat | 1:500 | Abcam | ab106836 |
| Goat anti-rabbit IgG H&L (HRP) | Secondary | Goat | 1:1000 | Abcam | ab205718 |
| Donkey anti-goat IgG H&L (HRP) | Secondary | Donkey | 1:1000 | Abcam | ab97110 |
| Goat anti-mouse IgG H&L (HRP) | Secondary | Goat | 1:1000 | Abcam | ab205719 |

**Table S3.** Comparison of treatment P-values for longissimus muscle protein abundance obtained with pen as the experimental unit (primary analysis) and with individual steer as the experimental unit (sensitivity analysis).

| **Protein** | **CON¹** | **LUB¹** | **Pen-based P²** | **Steer-based P³** |
| --- | --- | --- | --- | --- |
| Total Akt | -0.12 | -0.45 | 0.52 | 0.42 |
| p-Akt | -0.63 | -0.05 | 0.15 | 0.11 |
| p-Akt/Akt | -0.51 | 0.40 | 0.17 | 0.08 |
| Total mTOR | 1.36 | 0.95 | 0.54 | 0.52 |
| p-mTOR | -0.23 | 0.90 | 0.03 | <0.01 |
| p-mTOR/mTOR | -1.58 | -0.05 | 0.05 | 0.02 |
| Total p70S6K | -0.56 | 0.04 | 0.26 | 0.08 |
| p-p70S6K | 0.67 | 0.46 | 0.80 | 0.71 |
| p-p70S6K/p70S6K | 1.23 | 0.42 | 0.21 | 0.13 |
| Total CREB | -2.00 | -0.97 | 0.25 | 0.07 |
| p-CREB | 1.40 | 2.10 | 0.32 | 0.22 |
| p-CREB/CREB | 3.40 | 3.07 | 0.82 | 0.71 |
| Total AMPK | -1.10 | 0.29 | 0.17 | 0.04 |
| p-AMPK | -0.92 | -1.25 | 0.67 | 0.50 |
| p-AMPK/AMPK | 0.17 | -0.92 | 0.33 | 0.09 |
| PGC-1α | -0.38 | -0.20 | 0.48 | 0.45 |
| IGF-1 | -0.04 | 1.88 | 0.01 | <0.01 |

¹Least-squares means of the change in relative protein abundance from day 0 to day 56 (Δ = day 56 − day 0); CON = control, LUB = lubabegron.

²Primary analysis. Pen was the experimental unit for treatment and individual steer the observational unit (n = 4 pens and 16 steers per treatment). Values are those reported in the manuscript, obtained from a linear mixed-effects model with treatment as a fixed effect and pen as a random effect, fitted by REML.

³Sensitivity analysis. Individual steer was treated as the experimental unit (n = 16 per treatment), ignoring the pen structure. This analysis is anticonservative because lubabegron was delivered in the total mixed ration at the pen level and steers within a pen are not independent replicates; it is presented only to show the influence of the choice of experimental unit.

Conclusions were consistent between the two analyses for 14 of 17 endpoints. The endpoints central to the proposed mechanism, p-mTOR, the p-mTOR/mTOR ratio, and local IGF-1 protein, were significant under both analyses. Total AMPK, total CREB, and total p70S6K were significant or trending only when the pen structure was ignored, and these endpoints are reported as non-significant in the manuscript.
